# Host cell-dependent late entry step as determinant of hepatitis B virus infection

**DOI:** 10.1371/journal.ppat.1010633

**Published:** 2022-06-17

**Authors:** Xupeng Hong, Yuka Imamura Kawasawa, Stephan Menne, Jianming Hu

**Affiliations:** 1 Department of Microbiology and Immunology, The Pennsylvania State University College of Medicine, Hershey, Pennsylvania, United States of America; 2 Department of Pharmacology, Department of Biochemistry and Molecular Biology, Institute for Personalized Medicine, The Pennsylvania State University College of Medicine, Hershey, Pennsylvania, United States of America; 3 Department of Microbiology and Immunology, Georgetown University Medical Center, Washington, District of Columbia, United States of America; University of California, San Diego, UNITED STATES

## Abstract

Hepatitis B virus (HBV) has a highly restricted host range and cell tropism. Other than the human sodium taurocholate cotransporting polypeptide (huNTCP), the HBV entry receptor, host determinants of HBV susceptibility are poorly understood. Woodchucks are naturally infected with woodchuck hepatitis virus (WHV), closely related to HBV, but not with HBV. Here, we investigated the capabilities of woodchuck hepatic and human non-hepatic cell lines to support HBV infection. DNA transfection assays indicated that all cells tested supported both HBV and WHV replication steps post entry, including the viral covalently closed circular DNA (cccDNA) formation, which is essential for establishing and sustaining infection. Ectopic expression of huNTCP rendered one, but not the other, woodchuck hepatic cell line and the non-hepatic human cell line competent to support productive HBV entry, defined here by cccDNA formation during *de novo* infection. All huNTCP-expressing cell lines tested became susceptible to infection with hepatitis D virus (HDV) that shares the same entry receptor and initial steps of entry with HBV, suggesting that a late entry/trafficking step(s) of HBV infection was defective in one of the two woodchuck cell lines. In addition, the non-susceptible woodchuck hepatic cell line became susceptible to HBV after fusion with human hepatic cells, suggesting the lack of a host cell-dependent factor(s) in these cells. Comparative transcriptomic analysis of the two woodchuck cell lines revealed widespread differences in gene expression in multiple biological processes that may contribute to HBV infection. In conclusion, other than huNTCP, neither human- nor hepatocyte-specific factors are essential for productive HBV entry. Furthermore, a late trafficking step(s) during HBV infection, following the shared entry steps with HDV and before cccDNA formation, is subject to host cell regulation and thus, a host determinant of HBV infection.

## Author summary

Fundamental studies on, and development of therapies against, chronic hepatitis B virus (HBV) infection, which inflicts hundreds of millions worldwide, are impeded by deficiencies in HBV-susceptible animal models. HBV displays a strict species and cell tropism that are not clearly understood. Here, by studying replication of HBV, and the related woodchuck hepatitis virus, in human and woodchuck hepatic or non-hepatic cells, we found that non-hepatic human cells and some woodchuck hepatic cells could support productive HBV entry after expression of the human cell receptor for HBV. Moreover, by studying the infection of hepatitis D virus, which shares the same entry receptor and initial steps of entry with HBV, we could narrow down a host determinant of HBV infection operating at a late entry/trafficking step(s). Our study thus provides new insights into determinants of HBV host tropism and facilitates the development of HBV-susceptible animal models.

## Introduction

Chronic hepatitis B (CHB) is a critical public health problem affecting approximately 296 million individuals worldwide and resulting in 820,000 deaths every year due to severe liver disease progression, including fibrosis, cirrhosis, and hepatocellular carcinoma (HCC) [1]. Hepatitis B virus (HBV), a prototypic member of *Hepadnaviridae*, is the causative agent for CHB. Current treatment options for CHB, including reverse-transcriptase inhibitors and interferons, rarely cure HBV infection, due to the persistence of the viral covalently closed circular DNA (cccDNA) genome in the nuclei of infected hepatocytes [2]. Development and timely translation of curative therapies to CHB patients is impeded by the lack of HBV-susceptible animal models [3, 4]. Thus, understanding the determinants of HBV host tropism is a prerequisite for the development of animal models that are susceptible to HBV infection and are urgently needed for the ongoing pursuit of an HBV cure.

HBV is an enveloped para-retrovirus with a small relaxed circular DNA (rcDNA) genome. HBV cccDNA, the molecular basis for successful infection, is converted from the rcDNA inside the incoming virion during infection or the rcDNA inside the cytoplasmic nucleocapsid (NC) via intracellular amplification [5]. The protein free (PF) rcDNA, from which the reverse transcriptase attached to the 5’ end of the minus strand of rcDNA has been removed, accumulates in established cell lines and some PF-rcDNA may be an intermediate in cccDNA formation [6–9]. CccDNA exists as an episome in the nucleus of infected hepatocytes and serves as the template for the viral pregenomic RNA (pgRNA) and subgenomic RNAs [10]. Chimpanzees are the only non-human primates that are susceptible to HBV infection and that have served as a physiologically relevant model to study HBV infection, but they are no longer available for research due to ethical concerns [3]. Following the discovery of the human sodium taurocholate cotransporting polypeptide (huNTCP) as an essential HBV entry receptor [11, 12], it has been shown that expression of huNTCP in primary macaque and pig hepatocytes supports HBV infection, while huNTCP expression in primary mouse, rat, and dog hepatocytes was unable to confer susceptibility to HBV infection [13]. Recently, an HBV-susceptible rhesus macaque model based on viral vector mediated expression of huNTCP in the liver has been reported [14], but it is currently unknown if HBV infection in this primate model progresses to CHB. Hepatitis D virus (HDV), an RNA satellite virus that uses HBV envelope proteins for dissemination, also exploits huNTCP as its entry receptor [11, 12]. HBV and HDV do not infect normal mouse hepatocytes [15]. However, mouse liver cell lines expressing huNTCP and huNTCP-transgenic mice support HDV infection but remain refractory to HBV infection [12, 13, 15–18]. Moreover, HBV transgenic mice support intracellular HBV replication steps including virion assembly and secretion but fail to form HBV cccDNA [19], indicating an additional block in cccDNA formation via the intracellular amplification pathway. Unlike normal mouse hepatocytes or other mouse liver cell lines, an immortalized mouse hepatocyte cell line, AML12, can support HBV cccDNA formation via intracellular amplification, and upon ectopic expression of huNTCP, supports cccDNA formation during *de novo* infection albeit at a low level [20–22].

On the other hand, previous studies, which used HBV antigen expression and/or secretion as readouts for successful HBV infection, indicated that non-hepatic cells (e.g., HeLa, HEK293, A549, U2OS, CHO, and Vero cells) expressing huNTCP cannot support HBV infection [15, 16, 23], although they can support HDV infection [15, 16]. It is well-known that liver specific or enriched transcription factors, which are required for HBV gene transcription, are critical determinants of HBV tissue tropism [24]. Thus, it remains unclear whether lack of HBV antigen expression and/or secretion in the huNTCP-expressing non-hepatic cells is due to the inability to establish HBV infection (i.e., supporting HBV cccDNA formation) or to support HBV cccDNA transcription. Therefore, the precise restriction step(s) of HBV infection in non-human hepatocytes or non-hepatic cells remains to be more clearly defined.

Peking ducks and Eastern woodchucks (*Marmota monax*) are infected by their species-specific hepadnaviruses, duck hepatitis B virus (DHBV) and woodchuck hepatitis virus (WHV), which have been used as surrogate models for studying human HBV [3]. WHV is closely related to HBV in genome structure and replication [25]. WHV infection in woodchucks closely resembles HBV infection in humans, causing age-dependent acute or chronic infection and disease progression to HCC, and its preclinical use is predictive of therapeutic efficacy of nucleos(t)ide analogs and innate immune modulators against HBV in patients [26]. Moreover, the woodchuck model is fully immunocompetent and supported by the recent identification of the genome and liver transcriptome [27–29]. However, due to the sequence differences between WHV and HBV, evaluation of novel therapeutic strategies, such as capsid assembly modulators or siRNA and gene-editing approaches that are specific to HBV, but not WHV, proteins or DNA/RNA, would be rather challenging in woodchucks [30, 31]. HBV does not infect primary woodchuck hepatocytes [32], and there is no evidence for HBV infection in woodchucks.

In this study, we aimed to define the potential restriction steps for HBV infection in woodchuck hepatic and human non-hepatic cells. We investigated the capability of woodchuck and human hepatic cell lines to support both HBV and WHV replication and found that intracellular viral replication events, including cccDNA amplification, takes place independent of the host species. Furthermore, ectopic expression of huNTCP rendered one woodchuck hepatic and one human non-hepatic cell line able to support productive HBV entry, defined here by the establishment of cccDNA as no definitive marker of productive entry prior to cccDNA formation during HBV infection is currently available. However, another woodchuck hepatic cell line failed to support productive HBV entry but could support intracellular HBV replication events upon transfection and HDV infection upon huNTCP expression, indicating that a late entry/trafficking step(s) during HBV infection is subjected to host cell regulation and determines HBV tropism. These results thus reveal new insights into host regulation of HBV infection and provide the basis for developing new HBV-susceptible animal models.

## Results

### Woodchuck hepatic cell lines supported HBV replication, cccDNA formation, and virion secretion after replicon transfection

**Fig 1:**
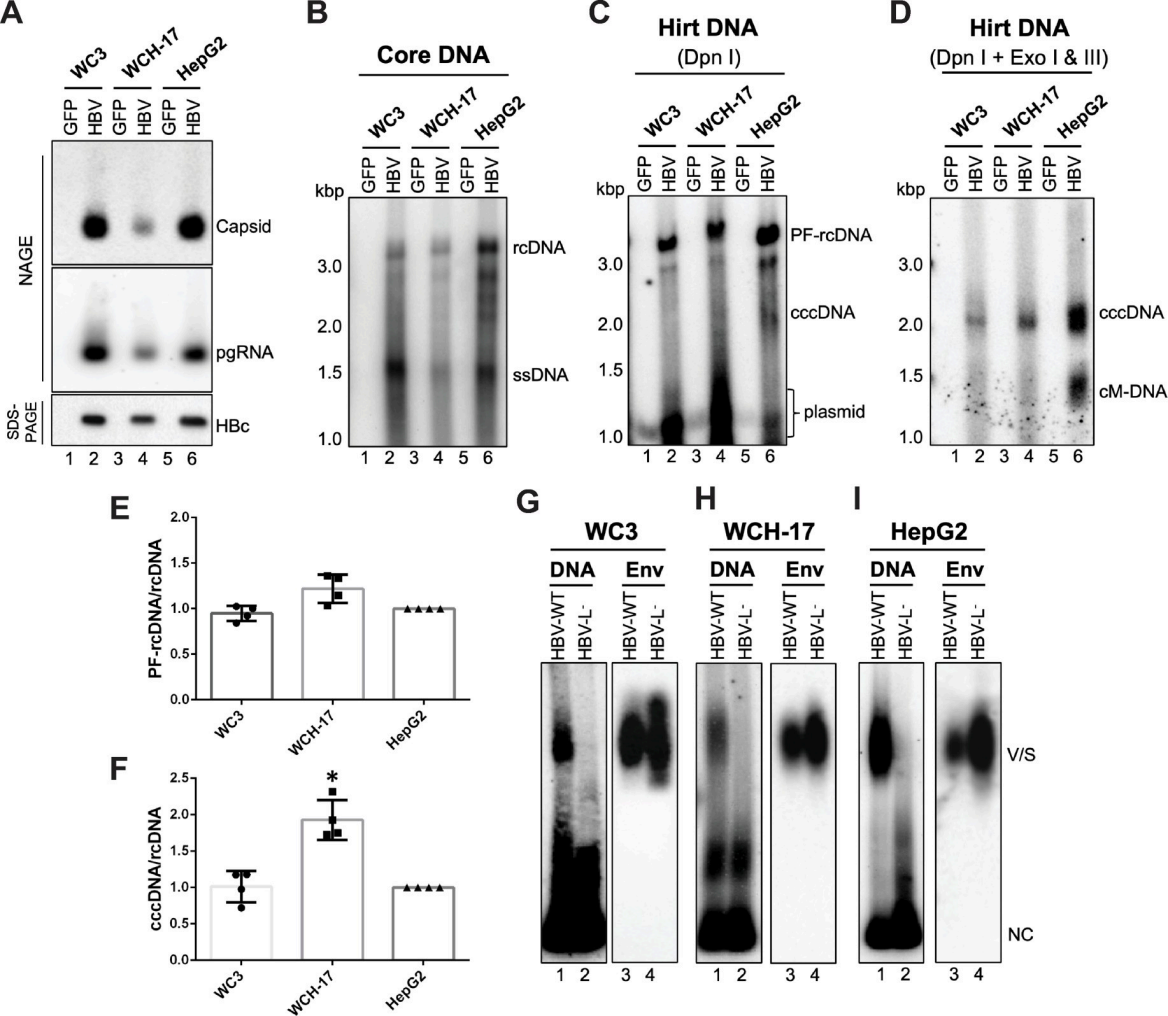
Woodchuck hepatic cell lines supported the HBV life cycle except entry. Woodchuck hepatic (hepatoma) cell lines WC3 and WCH-17 and human hepatoma cell line HepG2 were transfected with the HBV replicon (pCIΔA-HBV-HBc) or control (GFP) plasmids, and cells were harvested at 5 days post-transfection. (A) Cytoplasmic lysates were resolved by native agarose gel electrophoresis (NAGE) for comparing capsid assembly (top) and pgRNA packaging (middle) or by SDS-PAGE for comparing HBc expression (bottom). (B) Viral DNA inside nucleocapsids (i.e., core DNA) were released and detected by Southern blot analysis. One twentieth of total core DNA from transfected WC3 and WCH-17 cells and one thirtieth of total core DNA from the transfected HepG2 cells was loaded. PF DNA (i.e., Hirt DNA) was extracted from transfected cells by Hirt extraction method followed by Dpn I treatment to digest input plasmid (C) or with Dpn I and exonuclease I/III (Exo I/III) treatment to remove all DNA except closed circular DNA (D) before agarose gel electrophoresis and Southern blot analysis. One seventh of total WC3 Hirt DNA, one fourth of total WCH-17 Hirt DNA, and one tenth of total HepG2 Hirt DNA extracted from transfected cells was loaded. (E, F) PF-rcDNA and cccDNA signals from each cell line were normalized to the core rcDNA to compare the efficiencies on PF-rcDNA and cccDNA formation (means ± SD, n = 4). Statistical analysis was performed using student’s *t* test. *, *p* < 0.05. (G)-(I) Concentrated culture supernatant from transfected WC3 (G), WCH-17 (H), and HepG2 (I) cells were analyzed for virion and HBsAg subviral particle secretion by NAGE and detected by HBV DNA probe, followed by immunoblotting using an anti-HBs polyclonal antibody. ssDNA, single-strand DNA; cM-DNA, closed minus strand circular DNA; Env, envelope protein; V/S, virions and HBsAg subviral particles; NC, nucleocapsids.

WC3 and WCH-17 are two available woodchuck hepatic (hepatoma) cell lines [33, 34]. To explore whether these cell lines can support intracellular HBV replication, we transfected cells with the wild-type (WT) HBV replicon to bypass the entry step(s). We found that both cell lines supported HBV capsid assembly (**Fig 1A**, top), pgRNA packaging (**Fig 1A**, middle), and reverse transcription (**Fig 1B**), like the human hepatoma HepG2 cell line. To determine if these two woodchuck cell lines could support HBV cccDNA formation, we analyzed the Hirt DNA extracts from the replicon-transfected cells by pretreatment with Dpn I to remove the plasmid DNAs and then to detect the HBV PF-rcDNA and cccDNA by Southern blot analysis (**Fig 1C**). To further confirm cccDNA formation, we treated the Hirt DNA extracts with exonuclease I and III (Exo I/III) following Dpn I digestion to remove all DNA species except covalently closed circles [35]. This revealed the presence of HBV cccDNA in both WC3 and WCH-17 cells (**Fig 1D**), indicating that both woodchuck hepatic cell lines were competent for HBV cccDNA formation via the intracellular amplification pathway. When comparing the efficiencies of HBV cccDNA or PF-rcDNA formation from rcDNA between the human HepG2 cell line and the two woodchuck cell lines, we found that WCH-17 cells showed a slightly higher efficiency in HBV PF-rcDNA (1.22-fold) and cccDNA (1.93-fold) formation than WC3 cells, which were similar to HepG2 cells (**Fig 1E** and **1F**). These results suggested that host factors involved in HBV cccDNA formation are conserved between woodchucks and humans. We also investigated whether woodchuck hepatoma cells support HBV virion secretion after transfection with the HBV replicon. In contrast to cells transfected with an HBV replicon lacking the expression of L-HBs (HBV-L^-^), which is known to be deficient in virion secretion, we detected HBV virion secretion from both woodchuck cell lines transfected with the WT HBV replicon (HBV-WT) (**Fig 1G–1I**, lane 1 vs. 2). Furthermore, we found that the two woodchuck cell lines supported the secretion of HBs subviral particles (**Fig 1G–1I**, lanes 3,4). Altogether, our results indicated that woodchuck hepatoma cells, WC3 and WCH-17, support the entire HBV life cycle except the entry steps.

### WHV formed cccDNA in both human and woodchuck hepatoma cells via the intracellular amplification pathway

**Fig 2:**
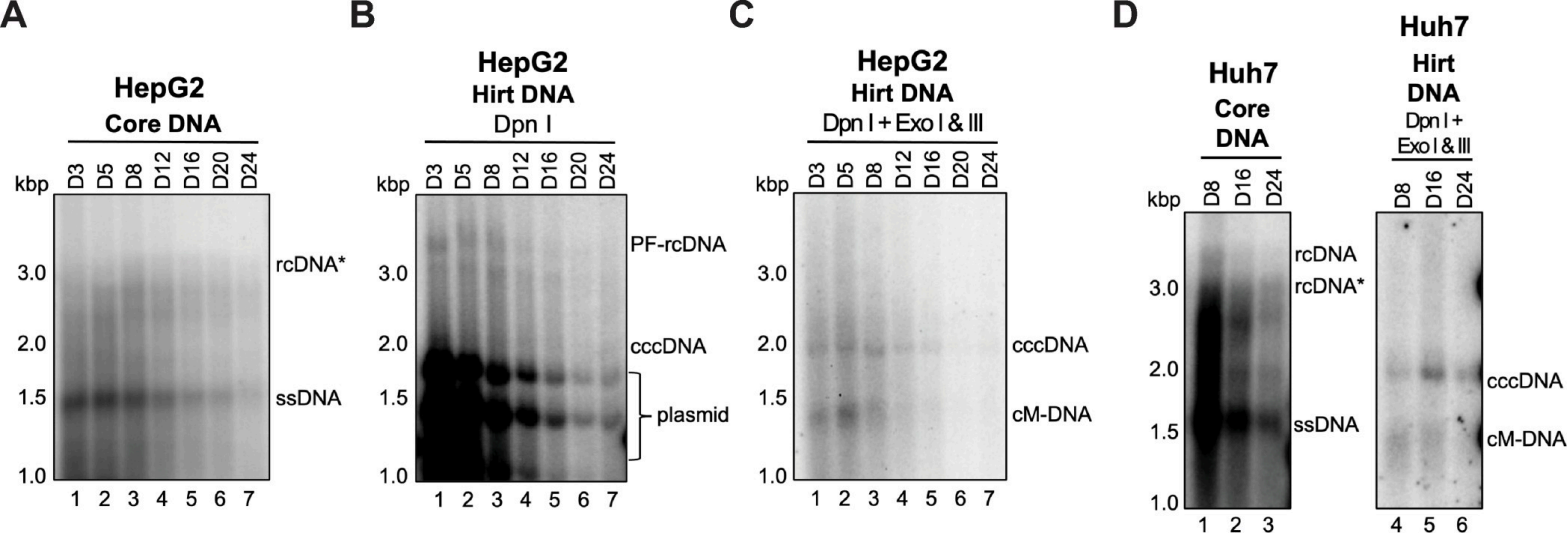
Human hepatic cell lines supported WHV cccDNA formation after transfection. Human hepatoma HepG2 cells were transfected with a WHV replicon, and cells were harvested at the indicated time points for analyzing core DNA (A) and Hirt DNA with Dpn I treatment (B) or with Dpn I plus Exo I/III treatment (C). The same experiment was repeated in another human hepatoma cell line Huh7 (D). ssDNA, single-strand DNA; rcDNA*, rcDNA with incomplete minus strand; cM-DNA, closed minus strand circular DNA.

Next, we asked whether human hepatic cells could support WHV replication and cccDNA formation. We transfected a WHV replicon into human or woodchuck hepatoma cells and analyzed viral DNA from transfected cells during a time-course post-transfection. In the human hepatoma HepG2 cells, we detected low levels of WHV rcDNA (**Fig 2A**), as well as WHV PF-rcDNA from Day 3 to 24 post-transfection (**Fig 2B**). Because two digested plasmid fragments containing WHV sequences comigrated and interfered with WHV cccDNA detection after DpnI treatment alone (**Fig 2B**), we treated the Hirt DNA extracts with both DpnI and Exo I/III and could then detect WHV cccDNA from Day 3 to 24 post-transfection in transfected HepG2 cells (**Fig 2C**). Our results thus clearly indicated that human hepatic cells support WHV cccDNA formation via the intracellular amplification pathway. We also showed that another human hepatoma cell line, Huh7, supported WHV cccDNA formation after WHV replicon transfection (**Fig 2D**). In contrast to obvious WHV rcDNA and PF-rcDNA detected in the human liver cell lines, we did not detect clear bands of WHV rcDNA and PF-rcDNA in WC3 cells by Southern blotting following WHV replicon transfection (**S1A** and **S1B Fig)**. However, we were able to detect low levels of WHV cccDNA in WC3 cells (**S1C Fig)**. Our results thus indicated that both human and woodchuck hepatic cells support WHV cccDNA formation via the intracellular amplification pathway. In agreement with a previous study [36], we could not detect WHV virion secretion from the replicon-transfected human or woodchuck hepatic cells (**S2A** and **S2B Fig**), even after cesium chloride (CsCl) density fractionation (**S2C Fig**), likely due to the very low expression of the WHV envelope proteins [36]. Interestingly, we observed that the DNA signals (i.e., DNA-containing NCs) in the WHV replicon-transfected cell culture supernatant migrated as a smear after the CsCl density fractionation, above the capsid protein signals (i.e., mostly empty capsids) (**S2C Fig**), suggesting that DNA-containing NCs might have aggregated and migrated slower than empty capsids after CsCl density ultracentrifugation. Together with the above observation for HBV cccDNA formation in woodchuck hepatic cells (**Fig 1**), our results indicated that host factors required for HBV or WHV cccDNA formation are conserved between humans and woodchucks, and these factors are interchangeable between the two host species to support cccDNA formation of the heterologous virus.

### Ectopic expression of huNTCP in woodchuck liver cells conferred susceptibility to HDV infection

**Fig 3:**
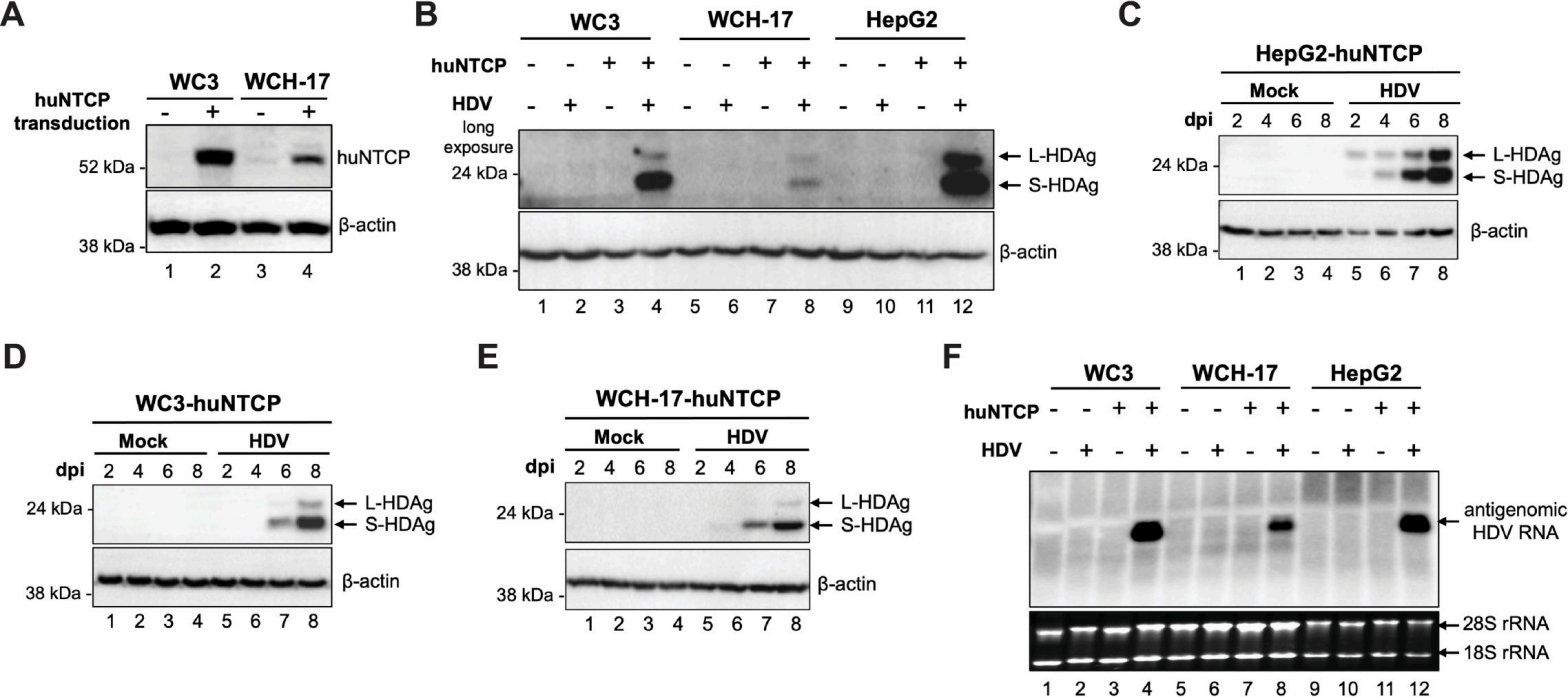
huNTCP expression in woodchuck hepatic cells conferred susceptibility to HDV infection. (A) Western blot analysis of deglycosylated cell lysates from parental and huNTCP-expressing cells for validating huNTCP expression. β-actin was used as the loading control. (B) Western blot analysis of whole cell lysates of parental and huNTCP-expressing cells at 8 days post-infection with HDV (ca. 200 genome equivalent per cell) for detecting HDAg using human HDAg antibody. Whole cell lysates of mock-infected or HDV-infected HepG2-huNTCP (C), WC3-huNTCP (D), and WCH-17-huNTCP (E) cells were harvested at 2-, 4-, 6-, and 8-days post-infection and resolved by SDS-PAGE and immunoblotted with human HDAg antibody. (F) Total RNA from mock- or HDV-infected parental or huNTCP-expressing cells were extracted at 8-days post-infection, and 5 μg of total RNA was analyzed by Northern blot for HDV antigenomic RNA. L-HDAg, large HDAg; S-HDAg, small HDAg.

Our cross-species transfection results raised the possibility that woodchuck and human hepatocytes could support heterologous virus infections after expression of the respective viral entry receptors. Whereas huNTCP is the known HBV entry receptor, the entry receptor for WHV has not been identified yet. Thus, we focused on exploring whether huNTCP is sufficient to allow HBV infection in woodchuck hepatic cells. We firstly generated woodchuck cell lines stably expressing huNTCP (i.e., WC3-huNTCP and WCH-17-huNTCP) by lentiviral transduction. We confirmed that both WC3-huNTCP and WCH-17-huNTCP cells expressed huNTCP (**Fig 3A**). Next, we infected the parental and huNTCP-expressing cells with HDV to determine if huNTCP expressed in WC3-huNTCP and WCH-17-huNTCP cells was functional to mediate HDV infection, since HDV shares with HBV the same entry receptor and early entry events. As a positive control, HepG2-huNTCP cells known to support HDV infection [11, 12] were used. We analyzed the HDV infection by western blotting for the hepatitis delta antigen (HDAg), which detected both the small and large HDAg isoforms, in a time dependent manner, in both huNTCP-expressing woodchuck hepatic cells and human HepG2 cells, but not in the parental cells (**Fig 3B–3E**). Furthermore, we could detect the HDV antigenomic RNA, the definitive proof of active HDV replication, by Northern blot analysis (**Fig 3F**). In agreement with the higher huNTCP expression level in WC3-huNTCP cells than in WCH-17-huNTCP cells (**Fig 3A**), we detected more efficient HDV infection in WC3-huNTCP cells than WCH-17-huNTCP cells as evidenced by higher levels of HDAg and HDV antigenomic RNA in WC3-huNTCP cells (**Fig 3B** and **3F**). In addition, immunofluorescence analysis of HDAg in the infected cells revealed ca. 5% of WCH-17-huNTCP cells and ca. 10% of WC3-huNTCP cells were HDAg-positive, consistent with the findings by the western blot and Northern blot analyses (**S3 Fig**). These results demonstrated that ectopic expression of huNTCP in WC3 and WCH-17 cells conferred susceptibility to HDV.

### The woodchuck hepatic cell line WCH-17, but not WC3, became susceptible to HBV infection after huNTCP expression

**Fig 4:**
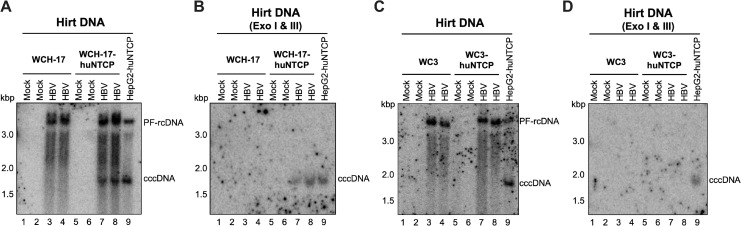
WCH-17 cells were rendered susceptible to HBV infection after huNTCP expression. WCH-17 (A, B) or WC3 (C, D) parental and huNTCP-expressing cells were plated on regular culture dishes (i.e., with no collagen coating) and infected with ca. 400 genome equivalent of HBV per cell. Three days post infection, the PF DNA (i.e., Hirt DNA) from mock- or HBV-infected cells was extracted by Hirt extraction and treated with Exo I/III followed by Southern blot analysis. Hirt DNA from HBV-infected HepG2-huNTCP cells, loaded at 4-fold less than the Hirt DNA from woodchuck cells, served as the positive control for cccDNA detection.

To determine if expression of huNTCP in woodchuck hepatic cells would support HBV infection, we firstly infected WC3-huNTCP and WCH-17-huNTCP cells with HBV at a multiplicity of infection (MOI) of 400 genome equivalent (GE) per cell and measured cccDNA formation at 3 days post-infection (dpi). As a positive control, HepG2-huNTCP cells were used (**Fig 4**). We detected HBV cccDNA in WCH-17-huNTCP cells but not in parental WCH-17 cells after HBV infection (**Figs 4A** and **S4**), which was further confirmed by Exo I/III treatment (**Figs 4B** and **S4**). Interestingly, unlike WCH-17-huNTCP, WC3-huNTCP cells failed to support HBV cccDNA formation during infection (**Figs 4C,4D** and **S4**), despite their capability to support HBV cccDNA formation after transfection of the HBV replicon (**Fig 1**). To further confirm that WC3-huNTCP cells were not susceptible to HBV infection, we increased the inoculum to an MOI of 2,000 GE/cell. Still, we were unable to detect cccDNA at either 3 dpi or 8 dpi (**S5 Fig**), suggesting that WC3-huNTCP cells could not support *de novo* cccDNA synthesis during infection. In addition, we harvested the infected cells after trypsinization to remove the virus bound to the cell surface and analyzed the Hirt DNA extracts, as a previous study indicated that PF-rcDNA is generated inside the cytoplasm during infection [37]. Consistently, we found that PF-rcDNA was barely detected from the inoculum but was increased until 2 dpi in HepG2-huNTCP cells (**S6 Fig**), suggesting that PF-rcDNA was indeed generated inside the cells after HBV internalization during infection. Therefore, PF-rcDNA could be used as a marker for HBV internalization. Noteworthily, in all the cell lines tested, huNTCP expression increased the levels of PF-rcDNA (**Figs 4** and **S4**), indicating that the expression of huNTCP increased HBV attachment and internalization, as reported by others [38]. Together with the fact that WC3-huNTCP cells could support HDV infection (**Fig 3**), at a level even higher than in WCH-17-huNTCP cells, these results indicated that WC3-huNTCP cells could support HBV attachment and internalization but failed to support *de novo* cccDNA synthesis. On the other hand, to exclude the possibility that huNTCP expression might somehow affect cccDNA formation in WC3-huNTCP cells, we transfected the HBV replicon into the huNTCP-expressing WC3 cells and found that these cells remained able to support HBV cccDNA formation via the intracellular amplification pathway (**S7 Fig**). Altogether, these results suggested that the block(s) to HBV cccDNA formation in WC3-huNTCP cells during infection occurs after huNTCP-mediated binding at the cell surface and the initial entry steps shared by both HBV and HDV but before nuclear import of rcDNA, a step common in cccDNA formation during infection and intracellular amplification (see also Discussion).

Since WCH-17 cells were competent to support HBV replication and secretion (**Fig 1**), and WCH-17-huNTCP cells were able to support HBV *de novo* cccDNA formation, we next tested whether WCH-17-huNTCP cells could support HBV gene expression from cccDNA after infection. We detected only very weak signals of the 3.5 kb pgRNA, 2.4 kb preS1/preS2/S mRNA, and 2.1 kb preS2/S mRNA by Northern blot analysis of RNA extracted from the infected WCH-17-huNTCP cells (**S8A Fig**). In addition, we could detect HBc-positive cells by immunofluorescence analysis (**S8B Fig**). According to Southern blot analysis, we estimated that 2x10^6^ copies of cccDNA were present in 1x10^7^ cells (**Figs 4** and **S4**). Assuming one cccDNA per infected cell at this early stage of infection [39, 40], we estimated that ca. 20% of WCH-17-huNTCP cells were infected. However, we could only detect HBc-positive WCH-17-huNTCP cells less than 1% frequency, which suggests that cccDNA transcription might not be efficient enough in most of the infected WCH-17-huNTCP cells to allow HBc detection by immunofluorescence analysis. To test this possibility, we transfected the replicon construct pHBV1.3 [41], which directs HBV RNA transcription using the native HBV promoters, in WC3, WCH-17, and HepG2 cells, and measured HBV RNA levels. Compared to the human hepatoma HepG2 cells, HBV pgRNA expressed by WC3 and WCH-17 cells transfected with pHBV1.3 were ca. 10–20 fold less (**S8C Fig**). On the other hand, the levels of the HBs RNAs (2.4 kb and 2.1 kb) and HBx RNA (0.7 kb) in the pHBV1.3-transfected WC3 and WCH-17 cells were ca. 3–7 fold lower than in the transfected HepG2 cells. As a control, we transfected these same cell lines with pCIΔA-HBV-HBc, which directs HBV core/pgRNA expression under the human cytomegalovirus (HCMV) immediate early promoter and HBs and HBx mRNAs from the native HBV promoters and that was used in all of the above transfection experiments to study HBV replication. The levels of pgRNA expressed from the strong and ubiquitously active HCMV promoter in this construct among the three cells lines were close to each other (within 2-fold difference). Furthermore, the HBs and HBx RNA levels expressed from this construct among the three cell lines were also close to each other (within 2-fold difference), suggesting that the strong HCMV enhancer (part of the HCMV promoter in pCIΔA-HBV-HBc) could also stimulate the native HBs and HBx promoters, which are the same in both pHBV1.3 and pCIΔA-HBV-HBc, to overcome their lower activity in the woodchuck cell lines relative to HepG2 cells. These results suggested that all HBV promoters were active in the woodchuck hepatic cells but at lower levels than in the human hepatic (HepG2) cells, especially the core (pgRNA) promoter. Altogether, our results suggested that huNTCP is the only human-specific factor that is required for productive HBV entry in WCH-17 cells, but the lower HBV promoter activity may limit HBV gene expression from cccDNA in the woodchuck cells. We attempted to test HBV infection in primary woodchuck hepatocytes with huNTCP expression *in vitro*; however, our efforts to isolate and culture differentiated primary woodchuck hepatocytes have not been successful for reasons yet to be elucidated.

### The human non-hepatic HEK293 cells became susceptible to HBV and HDV infection after huNTCP expression

**Fig 5:**
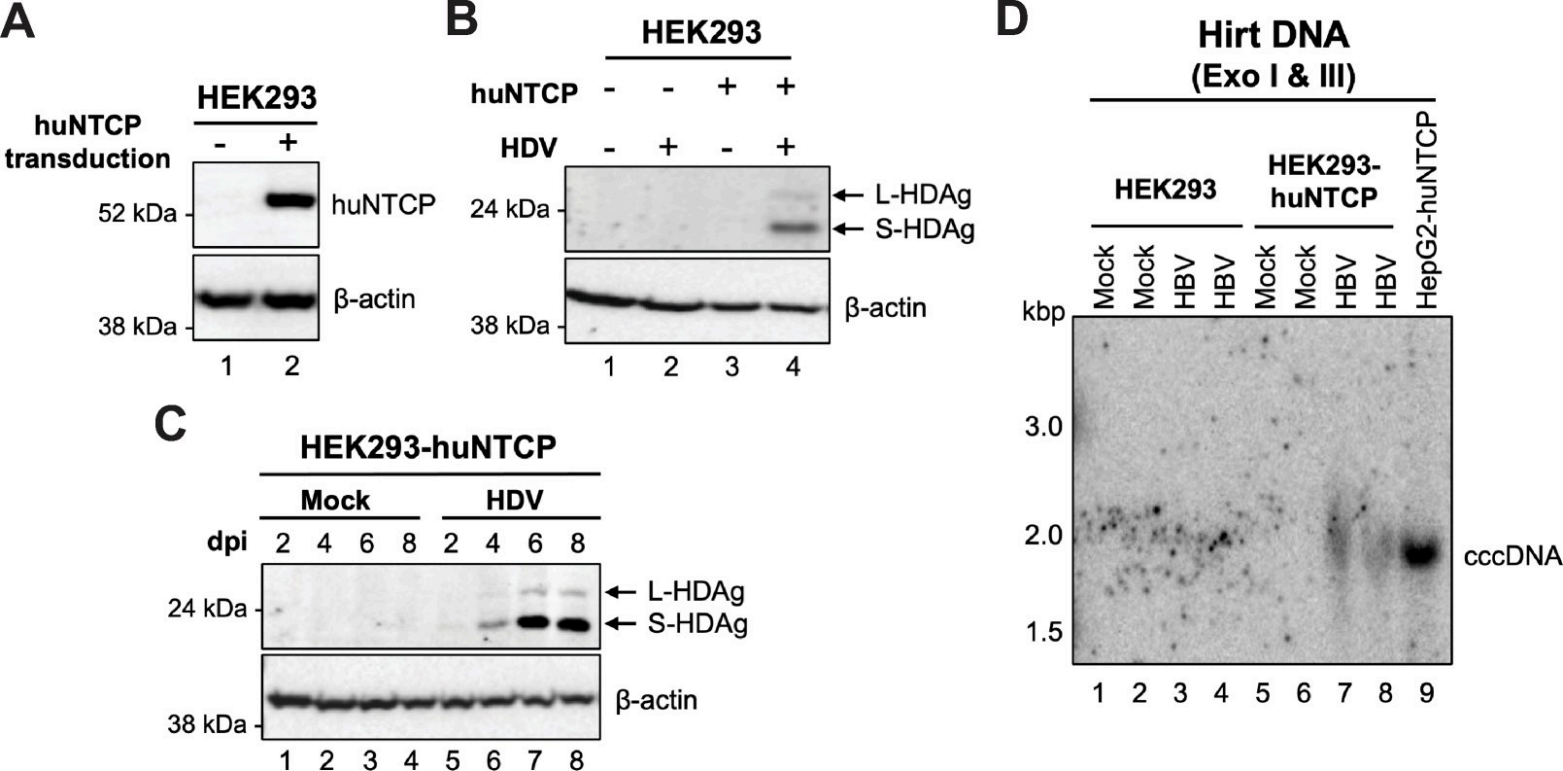
HEK293 cells were rendered susceptible to HBV and HDV infections after huNTCP expression. (A) Western blot analysis of deglycosylated cell lysates from parental and huNTCP-expressing HEK293 cells for validating huNTCP expression. β-actin was used as the loading control. (B) SDS-PAGE and western blot analysis of whole cell lysates of parental and huNTCP-expressing cells at 8 days post-infection with HDV for detecting HDAg using the human HDAg antibody. (C) SDS-PAGE and western blot analysis of whole cell lysates of mock-infected or HDV-infected huNTCP-expressing cells were harvested at 2-, 4-, 6-, and 8-days post-infection with the human HDAg antibody. (D) Parental and huNTCP-expressing HEK293 cells were plated on regular culture dishes (i.e., with no collagen coating) and infected with ca. 400 genome equivalent of HBV per cell. Three days post infection, the PF DNA (i.e., Hirt DNA) from mock- or HBV-infected cells was extracted by Hirt extraction and treated with Exo I/III followed by Southern blot analysis. Hirt DNA from HBV-infected HepG2-huNTCP cells, loaded at 4-fold less than the Hirt DNA from HEK293 and HEK-293-huNTCP cells, served as the positive control for cccDNA detection.

We previously reported that the human embryonic kidney cell line HEK293 supports HBV and DHBV cccDNA formation via intracellular amplification [8, 42]. However, others have reported that HEK293 cells failed to support productive HBV infection, as determined by the lack of detectable HBV antigen expression [23]. To test whether HEK293 cells indeed behave similar to WC3 cells, i.e., supporting cccDNA formation via intracellular amplification but not infection after huNTCP expression, we established huNTCP-expressing HEK293 cells (HEK293-huNTCP) by lentiviral transduction. We confirmed the expression of huNTCP by Western blot analysis (**Fig 5A**). We then infected both parental and huNTCP-expressing HEK293 cells with HDV to verify functionality of huNTCP in mediating viral entry steps shared by HDV and HBV. After HDV infection, we detected HDAg in HEK293-huNTCP cells but not in parental HEK293 cells at day 8 post-infection (**Fig 5B**), and the levels of HDAg were increased during the time-course in HEK293-huNTCP cells (**Fig 5C**), indicating that HEK293-huNTCP cells could support HDV infection. Next, we infected both HEK293 cells and HEK293-huNTCP cells with HBV and measured cccDNA formation. Strikingly, unlike WC3 cells (**Fig 4C** and **4D**), we detected HBV cccDNA in HEK293-huNTCP cells but not in parental HEK293 cells after HBV infection (**Fig 5D**), suggesting that hepatocyte-specific factors are not essential for *de novo* cccDNA formation during infection. Our results thus indicated that the human non-hepatic HEK293 cells could support HDV infection after huNTCP expression, and huNTCP is the only factor required for productive HBV entry (i.e., cccDNA formation) in HEK293 cells.

### WC3-huNTCP cells became susceptible to HBV infection after fusion with non-infectable HepG2 cells

To determine if WC3-huNTCP cells lack an essential facilitating factor(s) or express a dominant restriction (inhibitory) factor(s) for *de novo* cccDNA synthesis during infection, we performed a cell fusion experiment. The non-susceptible WC3-huNTCP cells were co-cultured with the non-susceptible WC3 or HepG2 cells, and cell fusion was induced by 50% PEG1500 for 5 min followed by HBV infection [16]. In contrast to the hybrid WC3-huNTCP and WC3 cells, which failed to support HBV infection, immunofluorescence analysis of the multinucleated hybrid cells formed between WC3-huNTCP and HepG2 cells revealed cells positive for HBc expression (**S9 Fig**), indicative of susceptibility to HBV infection. This result suggested that WC3-huNTCP cells lack an essential factor for *de novo* cccDNA formation. Furthermore, we detected HBc-positive multinucleated hybrid cells of HepG2-huNTCP cells fused with WC3 cells (**S9 Fig**), suggesting that WC3 cells do not express a dominant inhibitory factor for *de novo* cccDNA synthesis. Therefore, these results together indicated that the deficiency of cccDNA formation in WC3-huNTCP cells during infection was due to the lack of an essential facilitating factor rather than the existence of a dominant inhibitory factor.

### Comparative transcriptomic analysis of WC3 and WCH-17 cells revealed widespread differences

**Fig 6:**
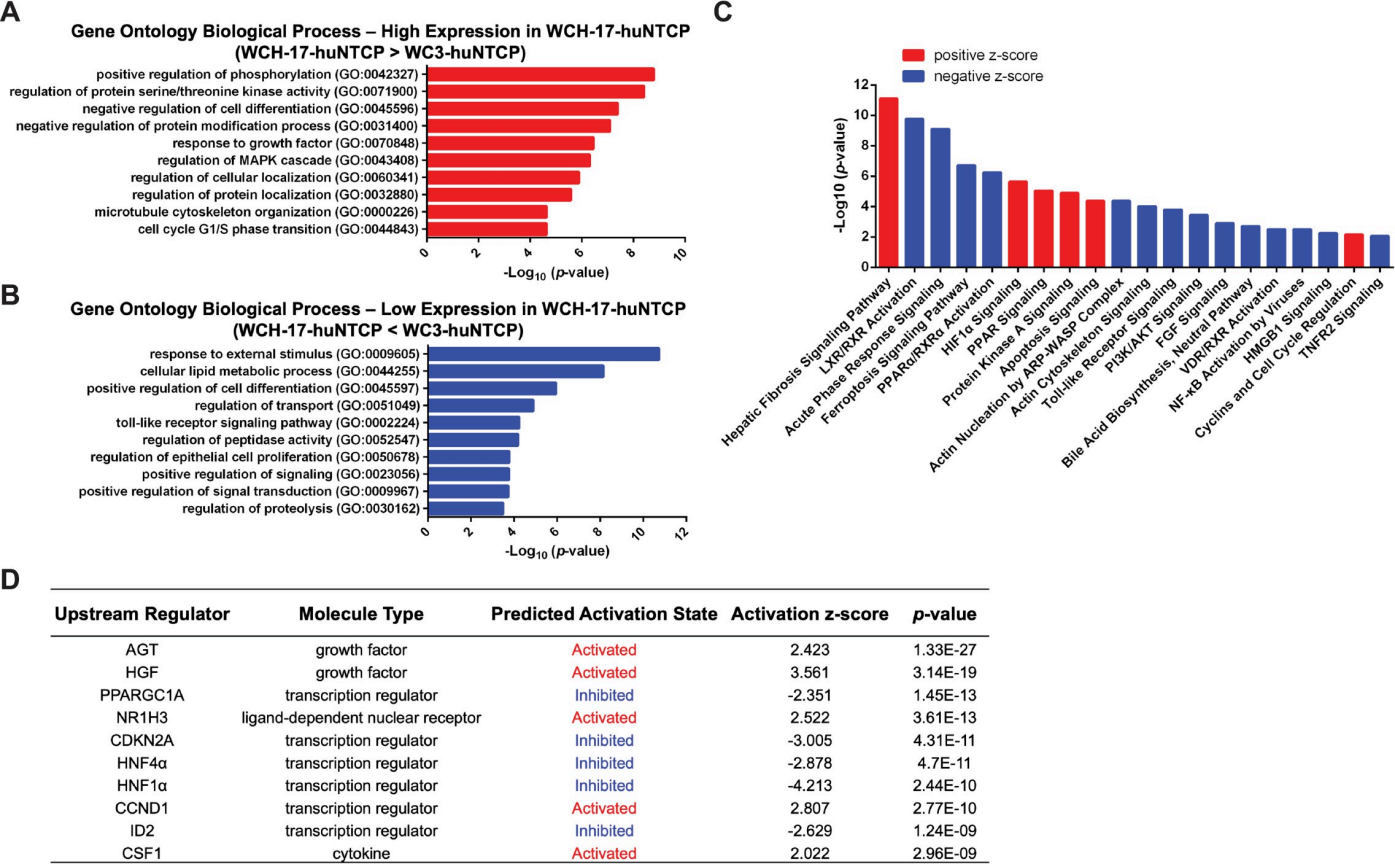
Comparative transcriptomic analysis of WC3 and WCH-17 huNTCP-expressing cells. (A) Gene Ontology (GO) analysis of biological processes for genes expressed at higher levels in WCH-17-huNTCP cells. (B) GO analysis of biological processes for genes expressed at lower levels in WCH-17-huNTCP cells. (C) The most highly regulated canonical pathways by IPA analysis of genes differentially expressed in WCH-17-huNTCP cells relative to WC3-huNTCP cells. Negative z-score (blue) or positive z-score (red) indicate the pathways that were likely inhibited or activated in WCH-17-huNTCP cells compared to WC3-huNTCP cells, respectively. (D) The top 10 upstream regulators and their predicted activation status in WCH-17-huNTCP cells (relative to WC3-huNTCP cells) in IPA analysis of transcriptomic results.

Since the above results indicated that WC3-huNTCP cells lack an essential factor for cccDNA formation during infection, we performed RNA-sequencing (RNA-seq) analysis to compare the transcriptomes of WC3-huNTCP and WCH-17-huNTCP cells as part of our initial efforts to identify potential factors that may be responsible for the difference in *de novo* cccDNA formation after infection between the two cell lines. We found widespread transcriptomic differences, with a total of 4841 genes differentially expressed between WCH-17-huNTCP and WC3-huNTCP cells (**S1 Dataset**), including 2396 genes expressed at higher levels and 2445 genes at lower levels in WCH17-huNTCP than in WC3-huNTCP cells (**S10A** and **S10B Fig**). Gene ontology analysis revealed that genes more highly expressed in WCH-17-huNTCP cells were significantly enriched in positive regulation of phosphorylation, negative regulation of cell differentiation, regulation of cellular localization, and microtubule cytoskeleton organization, etc. (**Fig 6A**). On the other hand, genes that were expressed at lower levels in WCH17-huNTCP cells showed significant enrichment in response to external stimulus, cellular lipid metabolic process, positive regulation of cell differentiation, and regulation of proteolysis, etc. (**Fig 6B**).

Differentially expressed genes between WC3-huNTCP and WCH-17-huNTCP were further analyzed by using Ingenuity Pathway Analysis (IPA), and the upstream regulatory pathways and regulators are shown in **S2** and **S3 Datasets**. In general, hepatocyte growth factor (HGF) and tricho-rhino-phalangeal syndrome type I protein (TRPS1) are two key regulators that were likely activated and inhibited, respectively, in WCH-17-huNTCP cells compared to WC3-huNTCP cells, and the differentially expressed genes were related to cell movement, invasion, transactivation, and development **(S10C Fig)**. Intriguingly, we found that the WC3-huNTCP and WCH-17-huNTCP cells showed significant differences in signaling pathways that are critical for hepatocyte functions, e.g., liver X nuclear receptor/retinoid X receptor (LXR/RXR) activation, peroxisome proliferator-activated receptor alpha/retinoid X receptor alpha (PPARα/RXRα) activation, and vitamin D receptor/ retinoid X receptor (VDR/RXR) activation (**Fig 6C** and **S2 Dataset**). When we analyzed the upstream regulators that were differentially expressed, we found that angiotensinogen (AGT), HGF, liver X nuclear receptor alpha (NR1H3), cyclin D1 (CCND1), and colony stimulating factor 1 (CSF1) were predicted to be activated, while PPARG coactivator 1 alpha (PPARGC1A), cyclin-dependent kinase inhibitor 2A (CDKN2A), hepatocyte nuclear factor 4α (HNF4α), hepatocyte nuclear factor 1α (HNF1α), and inhibitor of DNA binding 2 (ID2) were predicted to be inhibited in WCH-17-huNTCP as compared to WC3-huNTCP cells (**Fig 6D** and **S3 Dataset**). Furthermore, we compared liver-specific gene expression [43] between the two woodchuck hepatic cell lines, and found that 23 liver-specific genes (**S11A Fig**) and 40 genes related to liver development (**S11B Fig)** were differently expressed between WCH-17-huNTCP and WC3-huNTCP cells, suggesting that these two woodchuck hepatic cell lines have differences in hepatocyte differentiation and functions, which was also supported by the GO analysis and IPA (**Fig 6**).

The discrepancy of HBV cccDNA formation after infection and intracellular amplification in WC3 cells implies that rcDNA inside the nucleocapsids failed to be delivered into the nucleus specifically during infection, suggesting that an impeded intracellular transport of HBV may contribute to this failure. Indeed, we found that 186 genes associated with intracellular transport were differentially expressed between huNTCP-expressing WCH-17 and WC3 cells (**S4 Dataset)**. Especially, we found that 27 genes associated with endosomal transport, 24 genes with cytoskeleton-dependent transport, 16 genes with cytosolic transport, and 30 genes with nuclear transport were differentially expressed (**S12A–S12D Fig**). It is also possible that HBV was misdirected to the lysosome for degradation in WC3-huNTCP cells, leading to cccDNA formation failure. We found that 10 genes related to endosome to lysosome transport were differentially expressed (**S12E Fig**). Taken together, our transcriptomic analysis indicated that WC3 and WCH-17 cells had widespread differences, some of which may be responsible for the differential ability of these two woodchuck cell lines to support HBV infection after ectopic expression of huNTCP.

## Discussion

**Fig 7:**
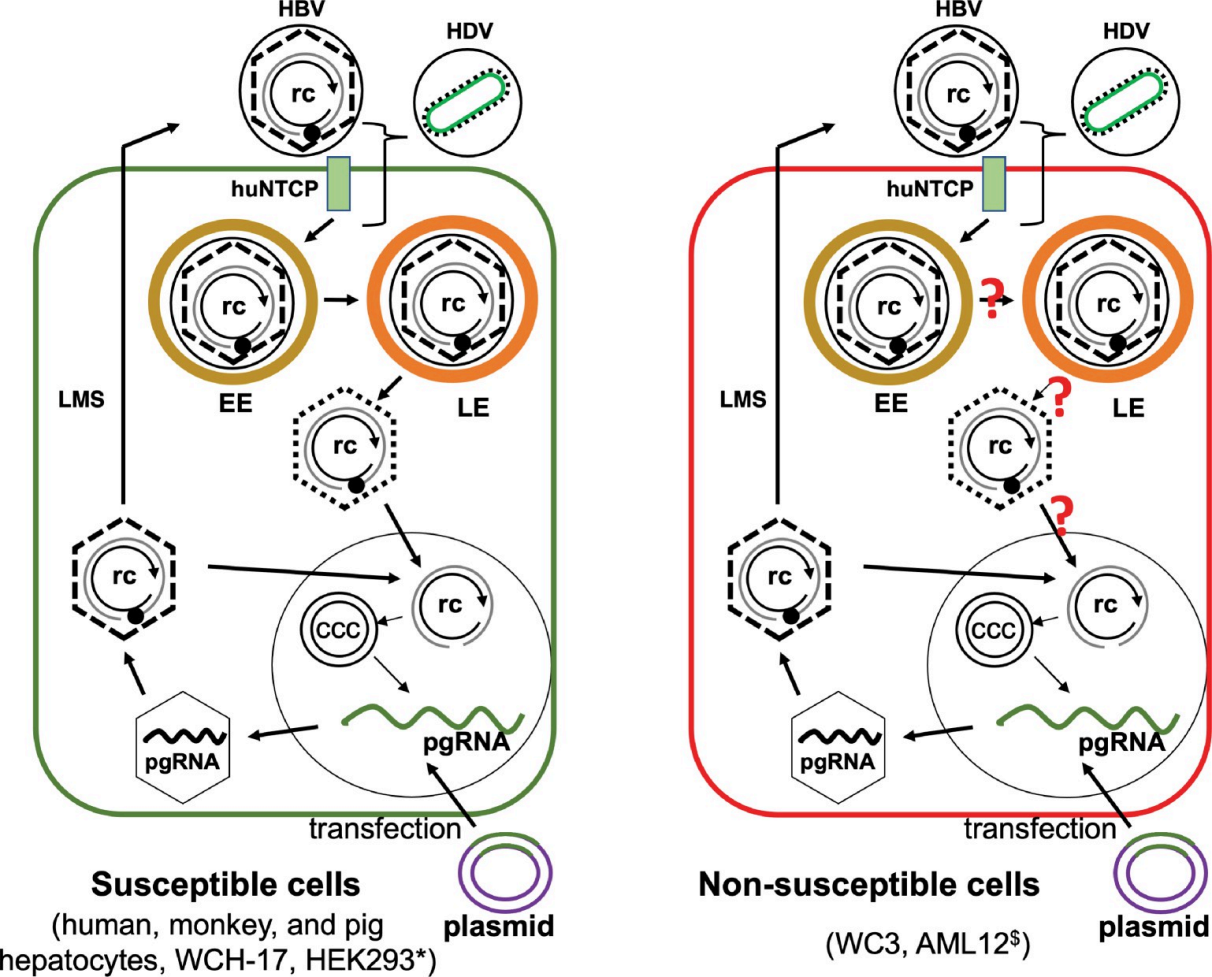
Model of HBV infection in susceptible and non-susceptible cells. WCH-17 and HEK293 (left) and WC3 (right) cells could support HBV cccDNA formation via the intracellular amplification pathway after HBV replicon transfection. Ectopic expression of huNTCP in WCH-17 and HEK293 cells (and other cells, see text) allowed HBV infection as evidenced by the establishment of cccDNA in the nucleus of infected cells. However, huNTCP expression in WC3 cells failed to support HBV infection, indicating an infection block at a step(s) after initial entry steps shared with HDV (in bracket) and prior to the nuclear import of rcDNA, the common step for cccDNA formation shared between infection and intracellular amplification. rc, relaxed circular DNA; ccc, covalently closed circular DNA; EE, early endosome; LE, late endosome; LMS, larger, middle, and small surface proteins. *, HEK293 cells cannot support HBV gene expression due to the lack of liver-specific transcription factors. ^$^, AML12 cells support efficient HBV cccDNA formation via intracellular amplification but are only weakly susceptible to HBV infection after huNTCP expression.?, potential steps during HBV infection that may be blocked in WC3 cells. See text for details.

HBV infection displays strict species and cell specificity. Here, we found that woodchuck hepatic cell lines could support HBV replication and in particular, cccDNA formation. Interestingly, both human and woodchuck hepatic cells could support replication of heterologous virus after cross-species transfection. Moreover, we found that huNTCP expression conferred susceptibility to HDV infection in woodchuck hepatic and human non-hepatic cell lines, but to HBV infection in only one of the woodchuck hepatic cell lines, WCH-17, and the human non-hepatic cell line, HEK293, suggesting that neither human- nor hepatocyte-specific factors are essential for establishing HBV infection, defined here as successful entry evidenced by the establishment of cccDNA. In contrast to its ability to support HBV cccDNA formation after transfection and the susceptibility to HDV infection, the woodchuck hepatic cell line WC3-huNTCP failed to support cccDNA formation during HBV infection, indicating a host cell-dependent blockade(s) during HBV infection after huNTCP-mediated binding at the cell surface and initial entry steps shared by both HBV and HDV but before nuclear import of rcDNA, a common step needed for cccDNA formation both during infection and intracellular amplification (**Fig 7**). Furthermore, WC3-huNTCP became susceptible to HBV infection after fusion with HepG2 cells, suggesting that WC3-huNTCP cells lack an essential factor required for cccDNA formation during infection. Our transcriptomic analysis of these two woodchuck hepatic cell lines revealed widespread differences in gene expression associated with intracellular transport, which may contribute to the differential susceptibility to HBV infection between WCH-17-huNTCP and WC3-huNTCP cells. Furthermore, the susceptibility of WCH-17-huNTCP cells to HBV infection raises the possibility that woodchucks can be rendered susceptible to HBV infection upon intrahepatic expression of huNTCP. These results thus provide new insights into mechanisms of HBV tropism and the basis to explore the development of an HBV-susceptible woodchuck model through intrahepatic expression of huNTCP.

That woodchuck and human hepatic cell lines could support both HBV and WHV cccDNA formation indicates that host factors required for this critical process are conserved between woodchucks and humans. Previous studies also found that human and chicken hepatoma cell lines support HBV and DHBV cccDNA formation [6, 44]. Furthermore, HEK293 supports HBV and DHBV cccDNA formation via intracellular amplification [6, 8, 42]. Therefore, no human- or hepatocyte-specific factors are required for HBV cccDNA formation via the intracellular amplification pathway. Importantly, in this study, we detected HBV cccDNA in the huNTCP-expressing human non-hepatic HEK293 and woodchuck WCH-17 cells during infection, suggesting that neither human- nor hepatocyte-specific factors are required for HBV cccDNA formation via *de novo* infection. Of course, HEK293 cells remain unable to support productive HBV replication after cccDNA formation due to the lack of liver transcription factors that are essential for HBV cccDNA transcription [24, 45], which explains the inability of HEK293 cells to allow productive HBV replication after huNTCP expression in a recent study [23]. On the other hand, our results indicated that human-specific hepatocyte transcription factors are not essential determinants for HBV species tropism as all HBV promoters did show activity at varying degrees in the woodchuck hepatic cells. The lower promoter activity of the HBV promoters, especially the core/pgRNA promoter, in the woodchuck hepatic cells as compared to the human hepatic cells, may be overcome, by directed evolution or engineering of the promoter sequences, to allow HBV adaptation to use the woodchuck hepatocyte transcription factors more efficiently. Since the woodchuck WCH-17 cells became susceptible to HBV infection after ectopic expression of huNTCP and these cells were competent to support the HBV lifecycle, the entry receptor is the only missing human-specific factor that is essential for HBV infection in these cells. These results suggest that huNTCP expression in woodchucks, and possibly in other non-susceptible species, may render them susceptible to HBV infection. Indeed, rhesus macaque hepatocytes became susceptible to HBV infection after huNTCP transduction both *in vitro* and *in vivo* [13, 14]. Since WHV-infected woodchucks are a well-established animal model for studying HBV pathogenesis and evaluating antivirals against HBV, the possibility to infect woodchucks with HBV would significantly expand the utility of this valuable model system to facilitate the ongoing HBV cure efforts [2, 3]. Therefore, we believe that efforts to test if woodchucks can be infected with HBV upon huNTCP expression are highly warranted.

WC3 cells were able to support post-entry steps of HBV replication, including intracellular cccDNA amplification, but they were unable to support HBV infection after huNTCP expression. This, together with the facts that WC3-huNTCP cells supported HDV infection and HBV internalization indicate that there is a barrier(s) after the huNTCP-mediated initial entry events of HBV infection that are shared with HDV but presumably before the nuclear import of rcDNA, which is required for cccDNA formation via either infection or intracellular amplification (**Fig 7**). Unlike the woodchuck hepatic cell lines we tested, mouse hepatocytes fail to support cccDNA formation even via the intracellular amplification pathway, suggesting that nucleocapsid uncoating or nuclear import is another likely block to HBV infection in the mouse cells. The AML12 mouse hepatocyte cell line supports efficient HBV cccDNA formation via intracellular amplification [22], but HBV infection in AML12 cells expressing huNTCP remains inefficient [20], again implicating an intracellular block(s) to HBV infection present in these mouse hepatocytes. As mouse hepatocytes expressing huNTCP are susceptible to HDV infection [16, 17], these results thus also implicate a similar block in AML12 cells (and normal mouse hepatocytes) to HBV infection at an entry stage similar to that in WC3 cells. The precise nature of this late entry block remains to be clearly defined but it could involve cytosolic trafficking events of the HBV capsid upon virus-cell membrane fusion possibly at the late endosome (**Fig 7**) [46]. Similar to the differential capability of WC3 cells to support HBV cccDNA formation via intracellular amplification vs. infection, HBV capsid mutations, as well as core protein allosteric modulators, inhibit cccDNA formation via *de novo* infection but enhance cccDNA formation via intracellular amplification [47–50]. These results clearly indicate that the HBV capsid is a critical regulator of cccDNA formation and likely exerts its effects via interaction with host factors. Some of these host interactions with the HBV capsid may be critical in controlling intracellular transport of HBV during infection to ensure productive entry but are deficient in WC3-huNTCP cells and mouse hepatocytes.

Both WC3 and WCH-17 are woodchuck liver cell lines derived from hepatomas. WC3 is derived from a WHV-free woodchuck treated with diethylnitrosamine [33], whereas the origin of WCH-17 is not well documented [34]. WCH-17 is believed to have been derived from a WHV-induced hepatoma [51]. However, we did not detect WHV DNA in WCH-17 cells and, consistently, our transcriptomic analysis indicated no WHV gene transcription in WCH-17 cells. Both cell lines showed extensive expression of genes associated with hepatocyte functions [43], with WC3 showing higher levels of hepatic gene transcription than WCH-17, suggesting hepatocyte differentiation as a major difference between the two cell lines. On the other hand, the human hepatocyte-derived cell line, Huh7, remains less susceptible to HBV infection than the well-differentiated hepatoblastoma HepG2 cell line after huNTCP reconstitution [12]. Thus, susceptibility to HBV infection is not necessarily correlated with the degree of hepatocyte differentiation, consistent with the lack of requirement for a hepatocyte-specific factor in HBV entry beyond the receptor huNTCP. Incidentally, the woodchuck NTCP (wNTCP) was reported to support HBV and HDV infection at low levels [52]. We detected no wNTCP RNA in the WC3 and WCH-17 cells in our RNA-seq analysis, consistent with their non-susceptibility to either HBV or HDV infection. Finally, huNTCP expression was shown to alter HBV transcription [53], which may affect cccDNA intracellular amplification at later time points during infection. However, in this study, we focused on cccDNA formation at 3 dpi in the huNTCP-expressing woodchuck cells when cccDNA was generated from the incoming virions [37].

Although huNTCP was identified as a bona fide HBV receptor 10 years ago [11], the entry events of HBV infection are still not well-defined. The differential susceptibility of the two woodchuck hepatic cell lines, upon huNTCP expression, provides an opportunity to examine in more detail the potential host factors, beyond huNTCP, that are involved in these HBV entry events. Our results here reveal a block to HBV infection in WC3 cells that likely operates after the initial entry steps shared between HBV and HDV but prior to the nuclear import of rcDNA. Since WC3-huNTCP cells fused with non-infectable HepG2 cells could support cccDNA formation during infection, WC3-huNTCP cells likely lack an essential host factor required for intracellular trafficking events specific to HBV, but not HDV, to allow *de novo* cccDNA formation. The HBV nucleocapsid may be released from the late endosome into the cytosol during infection [46]. Whether HDV is also released from the later endosome is currently unknown. Our RNA-seq analysis revealed that 186 genes related to intracellular transport (including endosomal, cytosolic, nuclear, etc.) were differentially expressed between WC3-huNTCP and WCH-17-huNTCP cells. Consistent with the assumption that endosomal transport may be involved in their differential susceptibility to HBV infection, 10 genes associated with endosome to lysosome transport were differentially expressed between WCH-17-huNTCP and WC3-huNTCP cells. Besides endosomal trafficking and release, it is also possible that the HBV nucleocapsid, after release into the cytosol, is transported differently during *de novo* infection than during intracellular cccDNA amplification [47, 54], and between susceptible and non-susceptible cells. HBV virion or nucleocapsid that is released into the cytosol during infection may be misdirected for degradation in non-susceptible cells. Our GO analysis indeed revealed that genes expressed at lower levels in WCH-17-huNTCP than WC3-huNTCP cells were significantly enriched in the regulation of peptidase activity and proteolysis. Interestingly, IPA indicated that LXR/RXR, PPARα/RXRα, and VDR/RXR activations were predicted to be inhibited in WCH-17-huNTCP cells compared to WC3-huNTCP cells. A recent study also showed that RXRα is an important host factor in modulating HBV infection, and the silencing of RXRα results in elevated HBV infection efficacy at an early stage [55]. Whether activation of these pathways is responsible for the non-susceptibility to HBV in WC3-huNTCP cells warrants further investigation. Furthermore, our transcriptomic data and IPA analysis also revealed that HNF1α, an important liver-enriched transcriptional factor, was predicted to be inhibited in WCH-17-huNTCP cells, whereas HNF1α was predicted to be activated in WC3-huNTCP cells. Unlike unmodified HBV-transgenic mice, HBV cccDNA could be detected in HBV transgenic mice deficient for HNF1α, albeit at a low level [56], raising the possibility that HNF1α is an upstream regulator that contributes to the block in HBV cccDNA formation in WC3-huNTCP cells during *de novo* HBV infection, as in mouse hepatocytes. The precise role and mechanism of HNF1α in HBV infection remain to be elucidated. HBV behaves as a “stealth virus”, which might evade the detection by pathogen recognition receptors after infection of hepatocytes [57, 58]. Our GO analysis found that genes expressed at lower levels in WCH-17-huNTCP cells were enriched in Toll-like receptor signaling, and IPA also indicated that NF-kB activation by virus was markedly inhibited in WCH-17-huNTCP cells, implying that innate immunity may contribute to the differential HBV susceptibility between WCH-17-huNTCP and WC3-huNTCP cells.

## Materials and methods

### Plasmids

pCIΔA-HBV-HBc (genotype D) [49], which directs HBV pgRNA expression under the human cytomegalovirus (HCMV) early gene expression promoter, is a wild-type plasmid replicon competent for HBV replication. Another HBV replication-competent plasmid (replicon) pHBV1.3, containing a 1.3-fold-overlength linearized HBV genome, directs HBV RNA transcription using native HBV promoters (a gift from Haitao Guo, University of Pittsburgh) [41]. pCIΔA-HBV-HBc-L^-^ is the HBV replicon plasmid that lacks large HBV surface protein (L-HBs) expression and thus does not support HBV virion secretion [50]. pCMV2-DC1X1.2 is a replication-competent construct of HDV (a gift from John Casey, Georgetown University) [59]. The HBV envelope expression plasmid LMS (genotype D, gtD) expressing large-, middle-, and small-HBs was constructed by removing the sequence including HCMV promoter sequence between SnaBI and BspEI on pCIΔA-HBV-HBc [45]. The wild-type WHV strain 8 (WHV8) replicon plasmid pucCMVWHV, directing WHV pgRNA under the control of HCMV immediate early promoter, was constructed and described in early studies [60, 61].

### Cells

Human hepatoma cell lines HepG2 and Huh7 cells, human embryonic kidney cell line HEK293, and woodchuck hepatoma cell lines WC3 (a gift from Haitao Guo) [33] and WCH-17 (CRL-2082, ATCC) [34] were cultured in Dulbecco’s modified Eagle’s medium (DMEM)-F12 supplemented with 10% fetal bovine serum (FBS) (Hyclone) and 50 μg/ml of penicillin-streptomycin as described previously [62, 63]. HepG2-huNTCP cells, which stably express huNTCP, were cultured as previously reported [50, 64].

### Transient transfection

Transient transfections of HepG2, Huh7, and WC3 were done as previously described [62, 63, 65]. For transfection in WCH-17 cells, cells were seeded in 60-mm dishes and transfected with 4 μg of plasmid using X-tremeGENE HP DNA Transfection Reagent (Roche). All transfection experiments were repeated between two to five times.

### Lentiviral transduction

Lentiviral particles encoding eGFP-tagged huNTCP (i.e., SLC10A1) and a puromycin resistance gene were purchased from GeneCopoeia (Maryland, USA). HEK293, WC3, and WCH-17 were transduced at MOI = 5 and selected under 5 μg/ml puromycin for stable huNTCP-expressing cell pools and the selection antibiotic was maintained during cell culturing.

### HBV and HDV infection

HBV virions were produced in HepAD38 culture (gtD) as described [35]. HDV was produced by co-transfection of pCMV2-DC1X1.2 and the HBV envelope protein construct LMS-gtD in Huh7 cells and cell culture supernatants were collected at days 7, 9, and 11 post-transfection. Virions in the culture supernatant were collected and concentrated by polyethylene glycol (PEG) precipitation. Cells were inoculated with ca. 200, 400, or 2000 viral genome equivalents (GE) HBV per cell, or 100 or 200 GE HDV per cell as indicated in media containing 4% PEG 8000 for 16 hours at 37°C. Mock-infected cells were treated with media concentrated from untransfected culture supernatant in the same way as from transfected cells. All cells were cultured in maintenance medium containing 2% DMSO and the culture medium was collected and changed every 2 days until cell harvest.

### Western blot analysis

Cells were lysed with NP40 lysis buffer (50 mM Tris-HCl [pH 8.0], 1 mM EDTA, 1% NP40) containing the protease inhibitor cocktail (Roche) for collecting cytoplasmic lysates after a brief spin to remove nuclei. Total cell lysates were collected after lysing with sodium dodecyl sulfate (SDS) sampling buffer (100mM Tris-HCl [pH 6.8], 5% SDS, 20% glycerol, 0.2% bromophenol blue, 10% beta-mercaptoethanol). Cell lysates were separated using 12.5% SDS-polyacrylamide gel electrophoresis (PAGE) as described early [62, 63]. For huNTCP western blot, cell lysates were treated with PNGase F (New England Biology, MA) following the manufacturer’s instruction and a polyclonal rabbit antibody against huNTCP (Cat no. HPA042727) (Sigma-Aldrich) was used for detection. The HDV-specific human monoclonal antibody (mAb) clone HD-T1/39 (a gift from John Casey) was used for detecting HDAg [66, 67]. The mouse mAb clone T2221 (Cat no. 2AHC24) and clone C33 (Cat no. 2ZC33) specific for the HBV and WHV core were purchased from Tokyo Future Style [63]. The polyclonal rabbit antibody against HBsAg (Cat no. 1811) (Virostat, Portland, ME, USA) was used for the detection of HBV surface proteins. The rabbit anti-β-actin antibody (Cat no. 4967S) (Cell Signaling Technology, ME, USA) was used as a loading control.

### Assay for capsid assembly and RNA packaging

To analyze the levels of assembled capsids and the amount of pgRNA packaged inside capsids, cytoplasmic NP40 lysates were resolved by 1% native agarose gel as described previously [62]. Following the transfer of viral particles from the gel to a nitrocellulose membrane, a ^32^P-labelled HBV anti-sense RNA riboprobe was used to detect encapsidated HBV RNA. Subsequently, the capsids were detected by the mouse mAb T2221 [62].

### Assay for virion secretion

Cell culture supernatant was precipitated with PEG 8000 as described [49]. The concentrated (by 50-fold) supernatant was digested with DNase I to remove plasmid DNAs and resolved by 1% native agarose gel as described previously [62]. Following the transfer of viral particles from the gel to a nitrocellulose membrane, a ^32^P-labelled HBV or WHV DNA probe was used to detect HBV or WHV DNA in the viral particles. Subsequently, the HBc or surface proteins were detected by the indicated core- or surface-specific antibodies as described previously [62].

### Southern blot analysis for viral DNA

The HBV or WHV core DNAs were released from viral nucleocapsids in cytoplasmic lysate by treatment with 0.5% SDS and 0.6 mg/ml Protease K (Invitrogen) at 37°C for 1 hour [49]. Hirt DNA extraction was employed for extracting PF DNAs [49]. Dpn I digestion was used to remove the input plasmids from HBV or WHV PF DNA in replicon-transfected cells. Then, the Dpn I-treated DNA from transfected cells or PF DNAs from HBV-infected cells were digested with Exonuclease I and III (Exo I and Exo III) as described [35, 49]. Viral DNAs were resolved on 1.2% agarose gel and detected by ^32^P-labeled HBV or WHV DNA probes.

### Northern blot analysis for HDV or HBV RNA

Total RNA from HDV-infected or HBV-infected cells (8 days post-infection) was extracted using Trizol (Life Technologies) or the Direct-zol RNA miniprep kit (Zymo Research, USA). Five μg, 10 μg, 15 μg, or 30 μg of total RNA as indicated was denatured in formaldehyde loading buffer at 65°C for 15 min. Samples were run on a 1% formaldehyde agarose gel and transferred to a nylon membrane following the instructions of the NorthernMax kit (Invitrogen). The hybridization was performed with the ^32^P-labeled HDV genomic RNA or HBV specific antisense riboprobe for 16 hours at 65°C to detect the HDV antigenomic RNA or HBV RNAs.

### Cell fusion

PEG-mediated cell fusion was done as previously described with slight modifications [16]. In a 24-well plate, 1.5 x 10^5^ HepG2, WC3, or WCH-17 cells were co-seeded with 1.5 x 10^5^ WC3-huNTCP or HepG2-huNTCP cells in each well. The next day, when cells reached 90% to 100% confluence, the cells were washed with PBS once and 250 ul pre-warmed 50% PEG 1500 (Roche) was added. The cells were then incubated at 37°C for 5 min. Cells were subsequently washed five times with PBS and fresh medium was added. Eight hours post-fusion, cells were infected with HBV at a MOI of 2000 GE/cell.

### Immunofluorescence

Cells cultured on glass-bottom 12 well plates (Cellvis) or 24 well collagen-I coated plate (Biocoat, Corning) were washed with PBS three times. Cells were then fixed with 4% paraformaldehyde for 20 min and permeabilized with 0.3% Triton X-100 for 5 min at room-temperature. The cells were subsequently incubated with blocking buffer (3% bovine serum albumin (BSA), 0.3% Triton X-100, 10% FBS in PBS) for 1 hour at room-temperature and then stained with the rabbit polyclonal anti-HBV core antibody (Zeta corporation, Arcadia, CA, USA) diluted 1:100 or the HDV-specific human mAb clone HD-T1/39 (a gift from John Casey, [66, 67]) diluted 1: 200 in PBS containing 0.3% Triton X-100 and 3% BSA. After washing with PBS, bound antibodies were detected using Alexa-Fluor 594-conjugated goat anti-rabbit or anti-human secondary antibody (Life Technologies) diluted 1:1000 in PBS containing 0.3% Triton X-100 and 3% BSA. Nuclei were stained with 1μg/mL 4’, 6-diamidine-2-phenylindole (DAPI) (Life Technologies). Images were acquired on a C2+ confocal microscope system (Nikon).

### RNA-sequencing (RNA-seq) and analysis

Total RNA was extracted from cells using Trizol (Life Technologies) and DNase treated with Direct-zol RNA miniprep kit (Zymo Research) following the manufacturer’s instructions. Optical density values of extracted RNA were measured using NanoDrop (Thermo Scientific) to confirm an A260:A280 ratio above 1.9. RNA integration number (RIN) was measured using BioAnalyzer (Agilent Technologies) RNA 6000 Nano Kit. For RNA-seq, 1 μg of total RNA was used for mRNA sequencing libraries preparation using the Illumina Stranded mRNA Prep, Ligation kit (Illumina) as per the manufacturer’s instructions. The libraries were pooled together and sequenced on an Illumina NovaSeq6000 and run for 2X53 cycles according to the manufacturer’s instructions. Two biological replicates were analyzed for each cell line. De-multiplexed and adapter-trimmed sequencing reads were generated using Illumina bcl2fastq (released version 2.20.0.422) allowing no mismatches in the index read. BBDuk (https://jgi.doe.gov/data-and-tools/bbtools/bb-tools-user-guide/bbduk-guide/) was used to trim/filter low quality sequences using “qtrim = lr trimq = 10 maq = 10” option. HISAT2 (v2.1.0) was used for alignment of the reads to the woodchuck genome Woodchuck_1.0 (GCA_014533835.1) [29] applying—no-mixed and—no-discordant options. Gene-level count data were generated using HTSeq. EdgeR in Galaxy (https://galaxyproject.org) was used to fit the read counts to the negative binomial model along with generalized linear model (GLM) and differentially expressed genes were determined by the likelihood ratio test method. Significance was defined to be those with *q*-value < 0.05 calculated by the Benjamini-Hochberg method to control the false discovery rate (FDR) and log2 fold-change is greater than 1 or smaller than -1, respectively. Significant differentially expressed genes from the RNA-seq of WC3-huNTCP and WCH-17-huNTCP cells were further analyzed through the Ingenuity Pathway Analysis (IPA) (Qiagen). The RNA-seq data generated in this paper have been deposited in the Gene Expression Omnibus (GEO) database, http://www.ncbi.nlm.nih.gov/ (accession number: GSE179682).

### Statistical analysis

DNA signals from Southern blot analysis were detected by Sapphire Biomolecular Imager (Azure Biosystems) and quantified using the Image Lab system 6.0.1 (Bio-Rad), as described [50, 62]. Data were analyzed by using Prism 7.0 (GraphPad). Student’s t test, two-tailed and unpaired, was used when comparing two datasets, and the data were shown as mean ± standard deviation (mean ± SD). *p* < 0.05 was considered statistically significant.

## Supporting information

S1 Fig Woodchuck hepatic cell lines supported WHV replication and cccDNA formation after transfection. Woodchuck hepatoma WC3 cells were transfected with the WHV replicon and cells were harvested at the indicated time points. (A) Viral DNA inside nucleocapsids (i.e., core DNA) was released by SDS-proteinase K treatment and detected by Southern blot analysis. (B) PF DNA (i.e., Hirt DNA) was extracted from transfected cells and treated with Dpn I to digest input plasmid (B) or with Dpn I and Exo I/III treatment to remove all DNA except closed circular DNA (C) before agarose gel electrophoresis and Southern blot analysis. ssDNA, single-strand DNA. cccDNA, covalently closed circular DNA.
(TIF)

S2 Fig WHV virions were not detected in the WHV-replicon transfected cell culture supernatant. (A) Human (HepG2 and Huh7) or woodchuck (WC3 and WCH-17) hepatic cell lines were transfected with the WHV replicon, and the cell culture supernatant was collected at Day 3 (for WCH-17), Day 10 (for WC3), or Day 14 (for HepG2 and Huh7) post-transfections. Viral particles in concentrated supernatant were resolved by native agarose gel electrophoresis (NAGE) and detected with a WHV DNA probe. (B) Concentrated cell culture supernatant from transfected HepG2 cells was harvest at the indicated time points and analyzed by NAGE assay and detected with a WHV DNA probe. The serum sample from WHV-infected woodchucks (lane 1) served as the positive control for enveloped WHV virions, and the NP40 lysates from WHV-transfected HepG2 cells (lane 10) served as the control for naked nucleocapsids (i.e., no envelope). (C) Cell culture supernatant from WHV-transfected HepG2 cells were collected at Day 14 post-transfection and fractionated by CsCl gradient ultracentrifugation. Indicated fractions (fractions 14 to 22) were resolved by NAGE and detected with a WHV DNA probe followed by immunoblot with an anti-WHc antibody (clone C33) for detecting viral capsids. Fraction 17 is predicted to have the peak in WHV virions at a density of 1.258 g/cm^3^. V, virions; NC, nucleocapsids.
(TIF)

S3 Fig Immunofluorescence analysis of HDAg in woodchuck hepatic cells. WC3 or WCH-17 parental and huNTCP-expressing cells were plated on collagen I-coated 24-well plate and infected with ca. 100 genome equivalent HDV per cell. Immunofluorescence analysis of HDAg in mock- or HDV-infected cells was performed at 8 dpi (magnification 200X).
(TIF)

S4 Fig WCH-17 but not WC3 cells were susceptible to HBV infection after huNTCP expression. WC3, WCH-17, or HepG2 parental and huNTCP-expressing cells were plated on collagen I-coated dishes and infected with ca. 400 genome equivalent HBV per cell. Three days post infection, the PF DNA (i.e., Hirt DNA) from mock- or HBV-infected cells was isolated by Hirt extraction and detected by Southern blot analysis without (A) or after treatment with Exo I/III (B). Hirt DNA from HBV-infected HepG2-huNTCP cells was used as the positive control for cccDNA detection. Equal amounts of Hirt DNA were loaded from infected cells.
(TIF)

S5 Fig WC3-huNTCP cells failed to support HBV infection. WC3-huNTCP cells were plated on regular dishes and infected with an MOI of ca. 400 or ca. 2000 genome equivalent (GE) of HBV per cell or mock-infected (MOI = 0). Three- or eight-days post infection, the PF DNA (i.e., Hirt DNA) from mock- or HBV-infected cells was isolated by Hirt extraction and detected by Southern blot analysis before (lane 1–6) or after (lane 9–14) treatment with Exo I/III. Hirt DNA from HBV-infected (ca. 400 GE/cell) or mock infected HepG2-huNTCP cells, loaded at 4-fold less than the Hirt DNA from woodchuck cells, served as the positive or negative control for cccDNA detection (lane 7, 8, 15, 16).
(TIF)

S6 Fig PF-rcDNA was generated inside the HBV-infected cells. HepG2-huNTCP cells were infected with ca. 200 genome equivalent (GE) HBV per cell. The infected cells were harvested at the indicated time points by trypsinization and washed twice by PBS to remove all cell surface-bound virus. The PF DNA (i.e., Hirt DNA) was then isolated by Hirt extraction and detected by Southern blot analysis. The Hirt DNA from the inoculum or output (the inoculum collected after the overnight incubation with the HepG2-huNTCP cells) was extracted after mixing with mock-infected cells. Equal amounts of Hirt DNA were loaded from the inoculum, output, and infected cells. Mitochondrial DNA (mtDNA) was used as the loading control.
(TIF)

S7 Fig huNTCP-expressing woodchuck as well as human hepatic cells supported HBV cccDNA formation via the intracellular amplification pathway. huNTCP-expressing cells were transfected with the HBV replicon (pCIΔA-HBV-HBc) or control (GFP) plasmid, and cells were harvested 5 days post-transfection for analyzing core DNA (A) and Hirt DNA with Dpn I treatment (B) or with Dpn I plus Exo I/III treatment (C). DNA was resolved by agarose gel electrophoresis and detected by Southern blot analysis.
(TIF)

S8 Fig WCH-17-huNTCP cells could support low levels of HBV gene expression after infection. WCH-17-huNTCP and HepG2-huNTCP cells were infected with ca. 2000 genome equivalent (GE) HBV per cell. (A) Total RNA from mock- or HBV-infected WCH-17-huNTCP or HepG2-huNTCP cells was extracted at 8-days post-infection (dpi) and HBV RNAs were detected by northern blot analysis. Fifteen μg of total RNA from HepG2-huNTCP cells and 30 μg of total RNA from WCH-17-huNTCP cells were loaded. (B) Immunofluorescence analysis of HBc expression in HBV-infected WCH-17-huNTCP cells at 8 dpi (magnification 400X). (C) Woodchuck hepatic cell lines WC3 and WCH-17 and human hepatoma cell line HepG2 were transfected with the HBV replicon (pCIΔA-HBV-HBc or pHBV1.3) or control (GFP) plasmid. The cells were harvested at 5 days post-transfection. Total RNA from the transfected cells were extracted and HBV RNAs were detected by northern blot analysis. Ten μg of total RNA from WC3 and WCH-17 cells and 5 μg of total RNA from HepG2 cells were loaded. The relative levels of the various HBV RNA species in the different cell lines are indicated at the bottom, after normalizing to the levels of the respective RNA species in the transfected HepG2 cells that are set at 100%.
(TIF)

S9 Fig WC3-huNTCP cells gained susceptibility to HBV infection after fusion to HepG2 cells. WC3 or HepG2 cells were co-seeded with WC3-huNTCP cells for 16 hours in a 24-well plate and treated with 50% PEG 1500 for 5 min to induce fusion. Similarly, WC3 cells were co-seeded with HepG2-huNTCP cells and fusion was induced. At 8 hours post-fusion, the cells were infected with HBV at a MOI of 2000 GE/cell. Immunofluorescence analysis of HBc expression in heterokaryotic cells was performed at 7 days post-infection (magnification 400X). The white and yellow arrowheads indicate the nucleus from the WC3 (or WC3-huNTCP) and HepG2 (or HepG2-huNTCP) cells, respectively. Also note that the GFP signal is diffuse throughout the cell in the HepG2-huNTCP cells as it was expressed as a free protein via an internal ribosomal entry site from the same RNA expressing huNTCP [63] whereas it is mostly on the plasma membrane in the WC3-huNTCP cells as it was expressed as the huNTCP-GFP fusion protein.
(TIF)

S10 Fig Comparative transcriptomic analysis of WC3 and WCH-17 huNTCP-expressing cells. (A) Volcano plots of differentially expressed genes (DEGs) between WCH-17-huNTCP and WC3-huNTCP cells. (B) Heat map of DEGs between WCH-17-huNTCP and WC3-huNTCP (fold-change > 2, adjust *p* value < 0.05). (C) Graphic summary of IPA analysis of DEGs between WCH-17-huNTCP and WC3-huNTCP cells.
(TIF)

S11 Fig Liver-related genes were differentially expressed between WC3-huNTCP and WCH-17-huNTCP cells. (A) Heat map of liver-specific genes that were differentially expressed between WC3 and WCH-17 huNTCP-expressing cells. (B) Heat map of DEGs associated with liver development (GO: 0001889).
(TIF)

S12 Fig Differentially expressed genes associated with intracellular transport. Heat map of genes differentially expressed between WC3 and WCH-17 huNTCP-expressing cells that are associated with (A) endosomal transport (GO: 0016197), (B) cytoskeleton-dependent transport (GO: 0030705), (C) cytosolic transport (GO: 0016482), (D) nuclear transport (GO: 0051169), and (E) endosome to lysosome transport (GO: 0008333).
(TIF)

S1 Dataset Differentially expressed genes between WC3-huNTCP and WCH-17-huNTCP cells. (XLSX)

S2 Dataset IPA analysis of upstream regulator on differentially expressed genes between WC3-huNTCP and WCH-17-huNTCP cells. (XLSX)

S3 Dataset IPA analysis of canonical pathway on differentially expressed genes between WC3-huNTCP and WCH-17-huNTCP cells. (XLSX)

S4 Dataset List of genes differentially expressed between WC3 and WCH-17 huNTCP-expressing cells that are associated with intracellular transport (GO: 0046907). (XLSX)
