# Supplementary figures and images for: Host cell-dependent late entry step as determinant of hepatitis B virus infection
Source: PLoS Pathog. 2022 Jun 17;18(6):e1010633. doi: 10.1371/journal.ppat.1010633 (PMC9246237; doi:10.1371/journal.ppat.1010633)

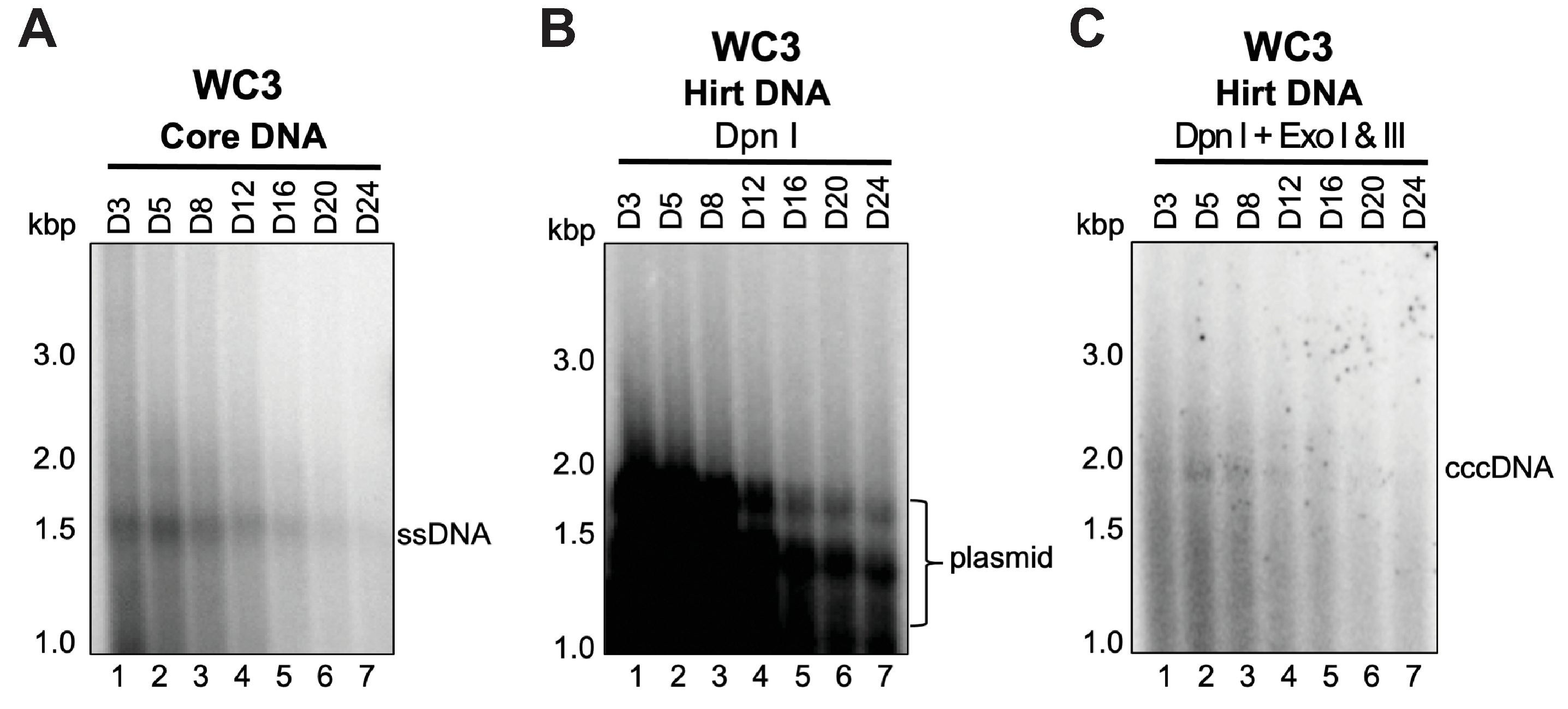

Supplement: S1 Fig — Woodchuck hepatoma WC3 cells were transfected with the WHV replicon and cells were harvested at the indicated time points. (A) Viral DNA inside nucleocapsids (i.e., core DNA) was released by SDS-proteinase K treatment and detected by Southern blot analysis. (B) PF DNA (i.e., Hirt DNA) was extracted from transfected cells and treated with Dpn I to digest input plasmid (B) or with Dpn I and Exo I/III treatment to remove all DNA except closed circular DNA (C) before agarose gel electrophoresis and Southern blot analysis. ssDNA, single-strand DNA. cccDNA, covalently closed circular DNA. (TIF) [file ppat.1010633.s001.tif]

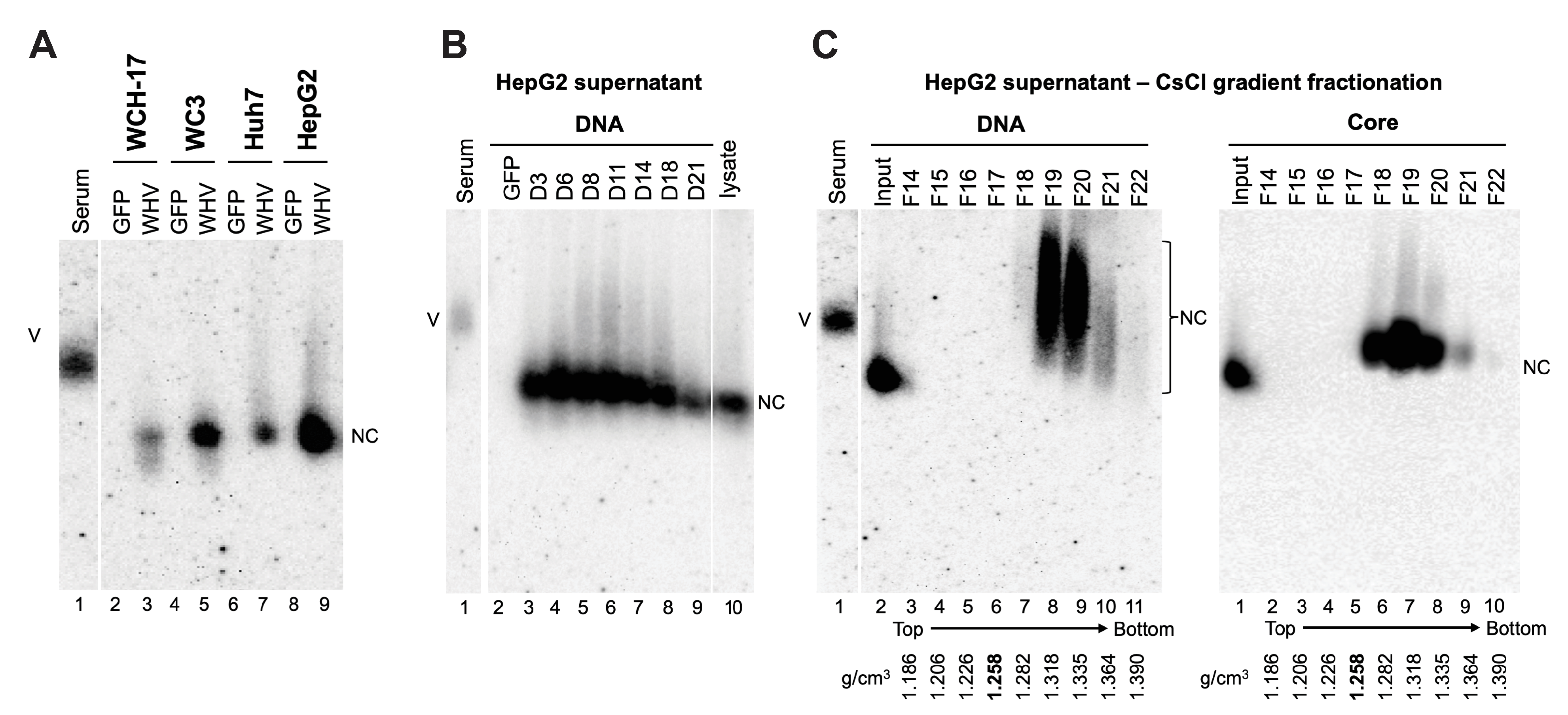

Supplement: S2 Fig — (A) Human (HepG2 and Huh7) or woodchuck (WC3 and WCH-17) hepatic cell lines were transfected with the WHV replicon, and the cell culture supernatant was collected at Day 3 (for WCH-17), Day 10 (for WC3), or Day 14 (for HepG2 and Huh7) post-transfections. Viral particles in concentrated supernatant were resolved by native agarose gel electrophoresis (NAGE) and detected with a WHV DNA probe. (B) Concentrated cell culture supernatant from transfected HepG2 cells was harvest at the indicated time points and analyzed by NAGE assay and detected with a WHV DNA probe. The serum sample from WHV-infected woodchucks (lane 1) served as the positive control for enveloped WHV virions, and the NP40 lysates from WHV-transfected HepG2 cells (lane 10) served as the control for naked nucleocapsids (i.e., no envelope). (C) Cell culture supernatant from WHV-transfected HepG2 cells were collected at Day 14 post-transfection and fractionated by CsCl gradient ultracentrifugation. Indicated fractions (fractions 14 to 22) were resolved by NAGE and detected with a WHV DNA probe followed by immunoblot with an anti-WHc antibody (clone C33) for detecting viral capsids. Fraction 17 is predicted to have the peak in WHV virions at a density of 1.258 g/cm3. V, virions; NC, nucleocapsids. (TIF) [file ppat.1010633.s002.tif]

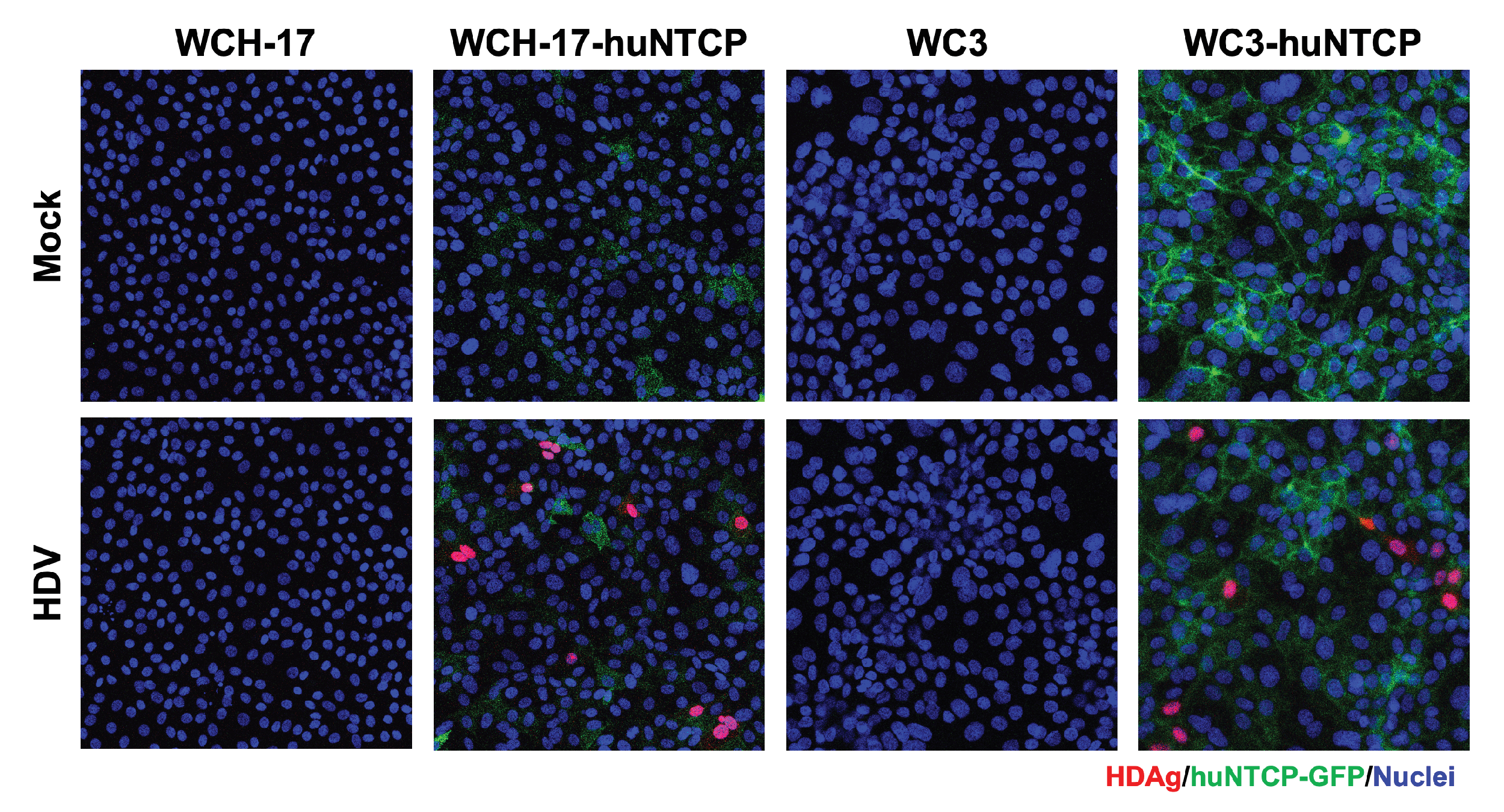

Supplement: S3 Fig — WC3 or WCH-17 parental and huNTCP-expressing cells were plated on collagen I-coated 24-well plate and infected with ca. 100 genome equivalent HDV per cell. Immunofluorescence analysis of HDAg in mock- or HDV-infected cells was performed at 8 dpi (magnification 200X). (TIF) [file ppat.1010633.s003.tif]

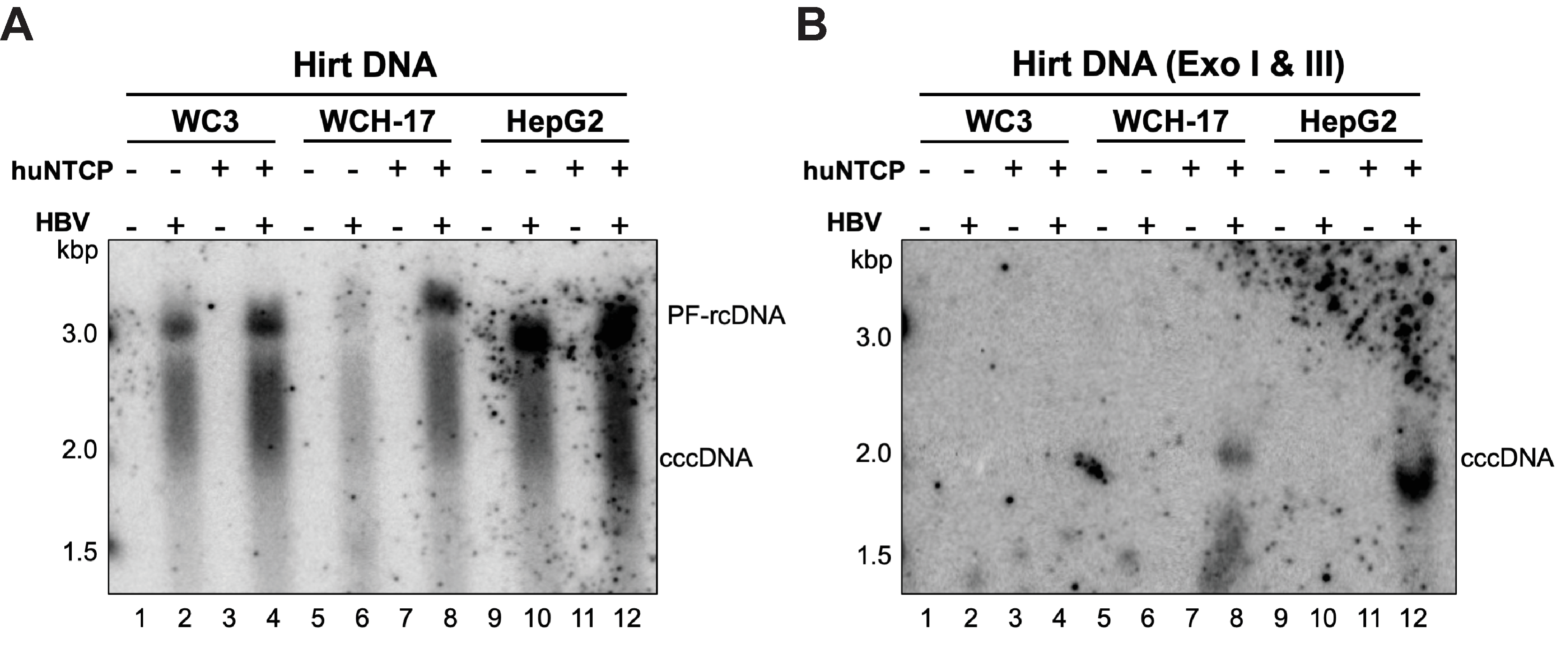

Supplement: S4 Fig — WC3, WCH-17, or HepG2 parental and huNTCP-expressing cells were plated on collagen I-coated dishes and infected with ca. 400 genome equivalent HBV per cell. Three days post infection, the PF DNA (i.e., Hirt DNA) from mock- or HBV-infected cells was isolated by Hirt extraction and detected by Southern blot analysis without (A) or after treatment with Exo I/III (B). Hirt DNA from HBV-infected HepG2-huNTCP cells was used as the positive control for cccDNA detection. Equal amounts of Hirt DNA were loaded from infected cells. (TIF) [file ppat.1010633.s004.tif]

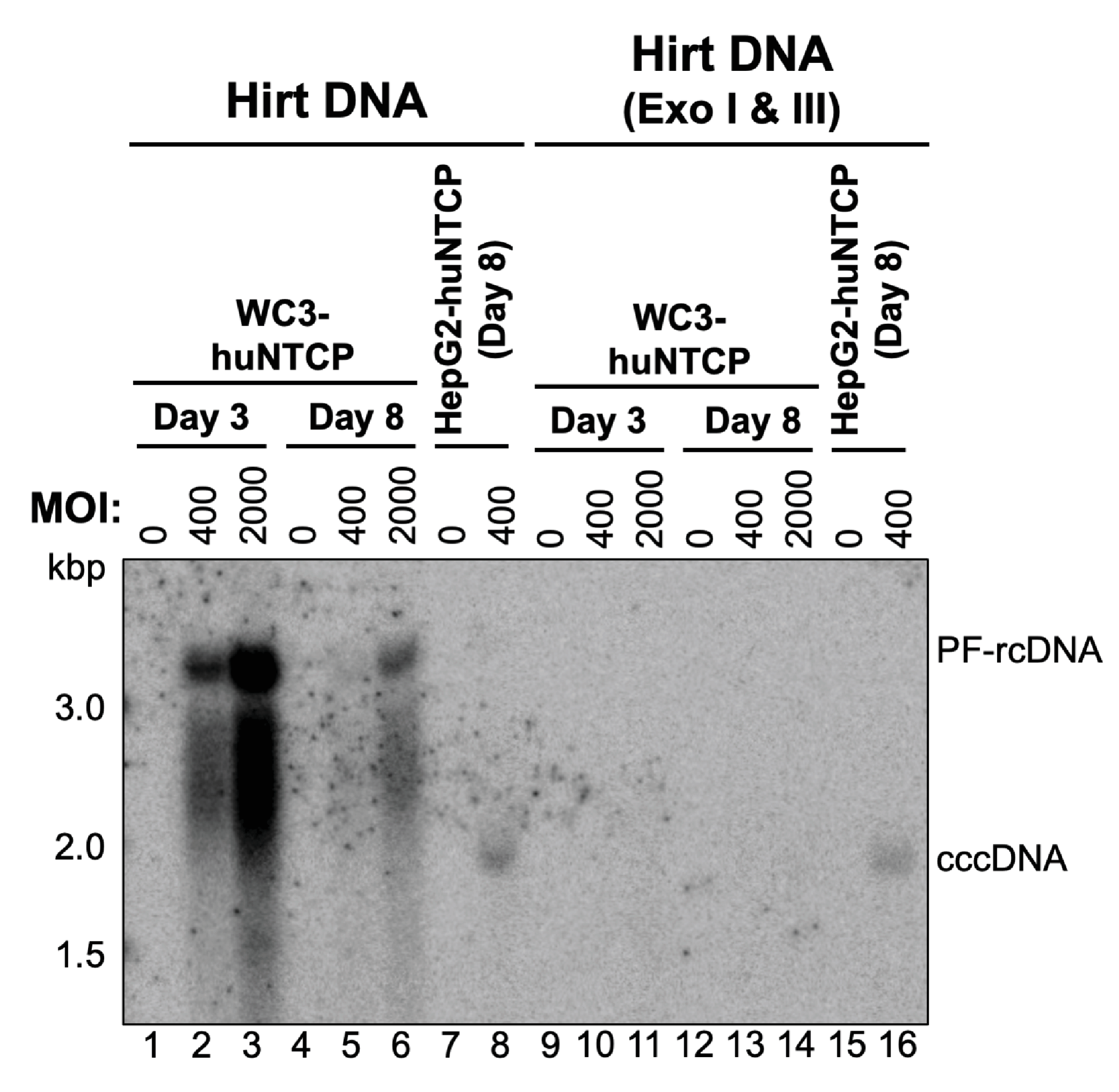

Supplement: S5 Fig — WC3-huNTCP cells were plated on regular dishes and infected with an MOI of ca. 400 or ca. 2000 genome equivalent (GE) of HBV per cell or mock-infected (MOI = 0). Three- or eight-days post infection, the PF DNA (i.e., Hirt DNA) from mock- or HBV-infected cells was isolated by Hirt extraction and detected by Southern blot analysis before (lane 1–6) or after (lane 9–14) treatment with Exo I/III. Hirt DNA from HBV-infected (ca. 400 GE/cell) or mock infected HepG2-huNTCP cells, loaded at 4-fold less than the Hirt DNA from woodchuck cells, served as the positive or negative control for cccDNA detection (lane 7, 8, 15, 16). (TIF) [file ppat.1010633.s005.tif]

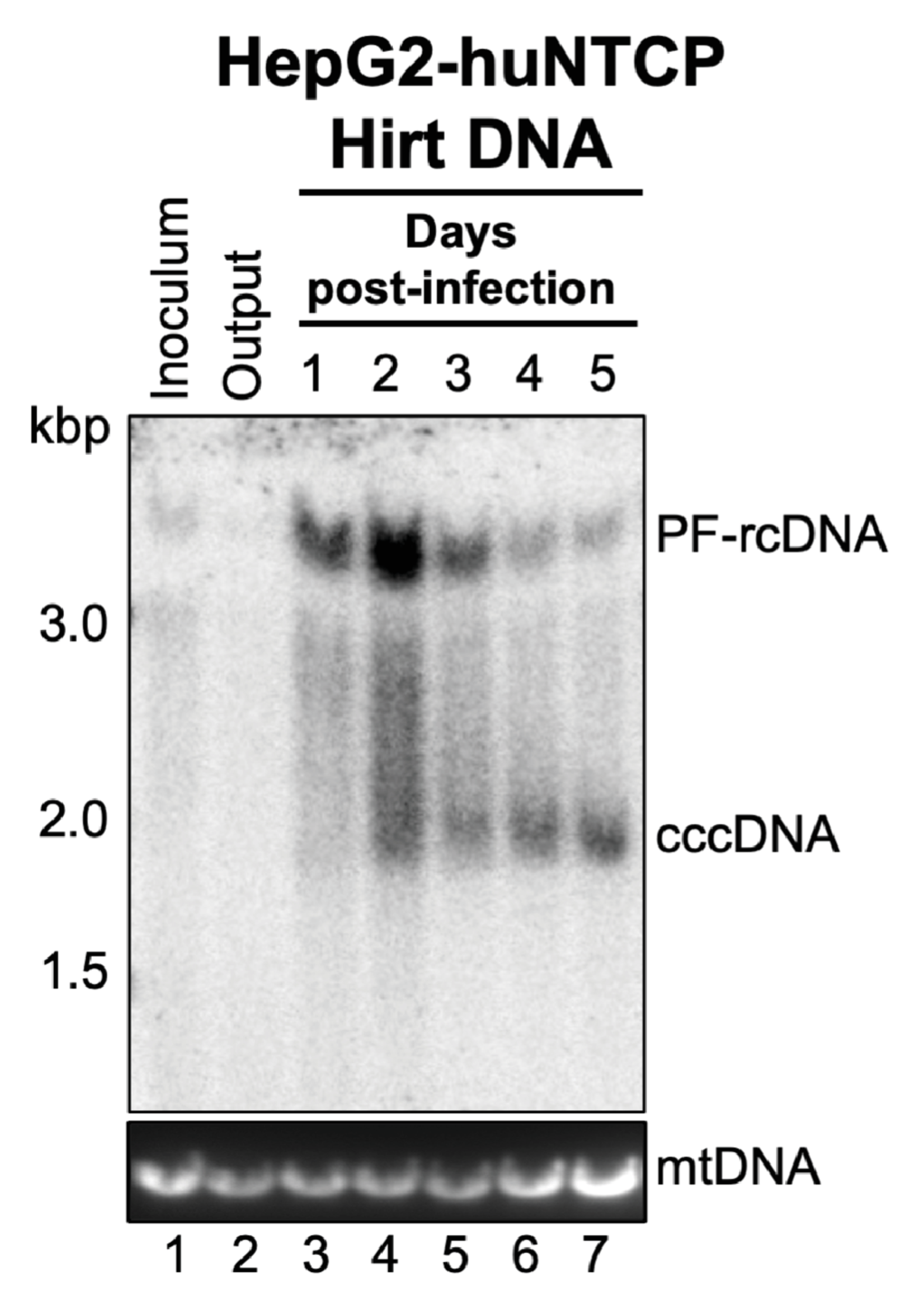

Supplement: S6 Fig — HepG2-huNTCP cells were infected with ca. 200 genome equivalent (GE) HBV per cell. The infected cells were harvested at the indicated time points by trypsinization and washed twice by PBS to remove all cell surface-bound virus. The PF DNA (i.e., Hirt DNA) was then isolated by Hirt extraction and detected by Southern blot analysis. The Hirt DNA from the inoculum or output (the inoculum collected after the overnight incubation with the HepG2-huNTCP cells) was extracted after mixing with mock-infected cells. Equal amounts of Hirt DNA were loaded from the inoculum, output, and infected cells. Mitochondrial DNA (mtDNA) was used as the loading control. (TIF) [file ppat.1010633.s006.tif]

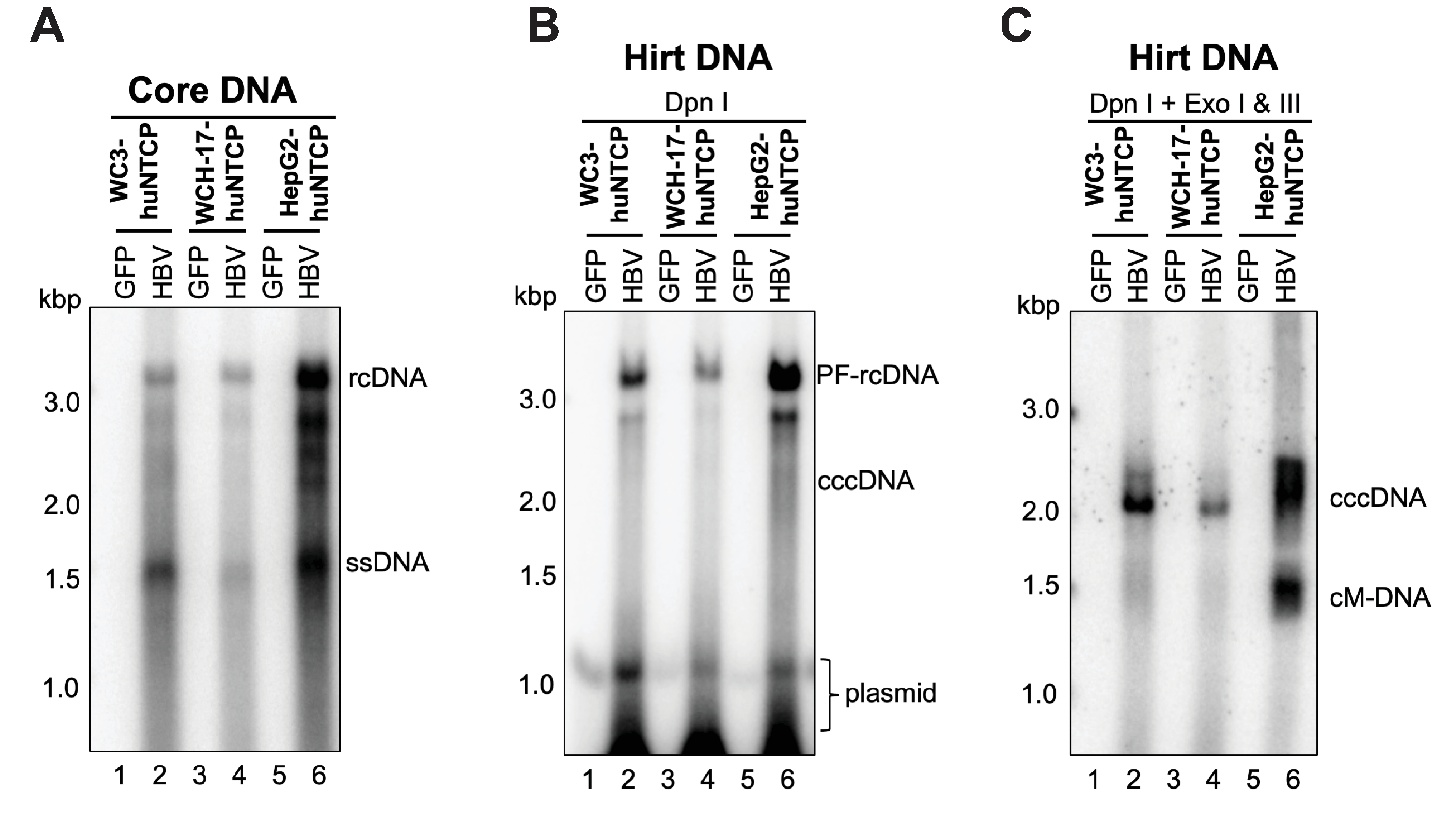

Supplement: S7 Fig — huNTCP-expressing cells were transfected with the HBV replicon (pCIΔA-HBV-HBc) or control (GFP) plasmid, and cells were harvested 5 days post-transfection for analyzing core DNA (A) and Hirt DNA with Dpn I treatment (B) or with Dpn I plus Exo I/III treatment (C). DNA was resolved by agarose gel electrophoresis and detected by Southern blot analysis. (TIF) [file ppat.1010633.s007.tif]

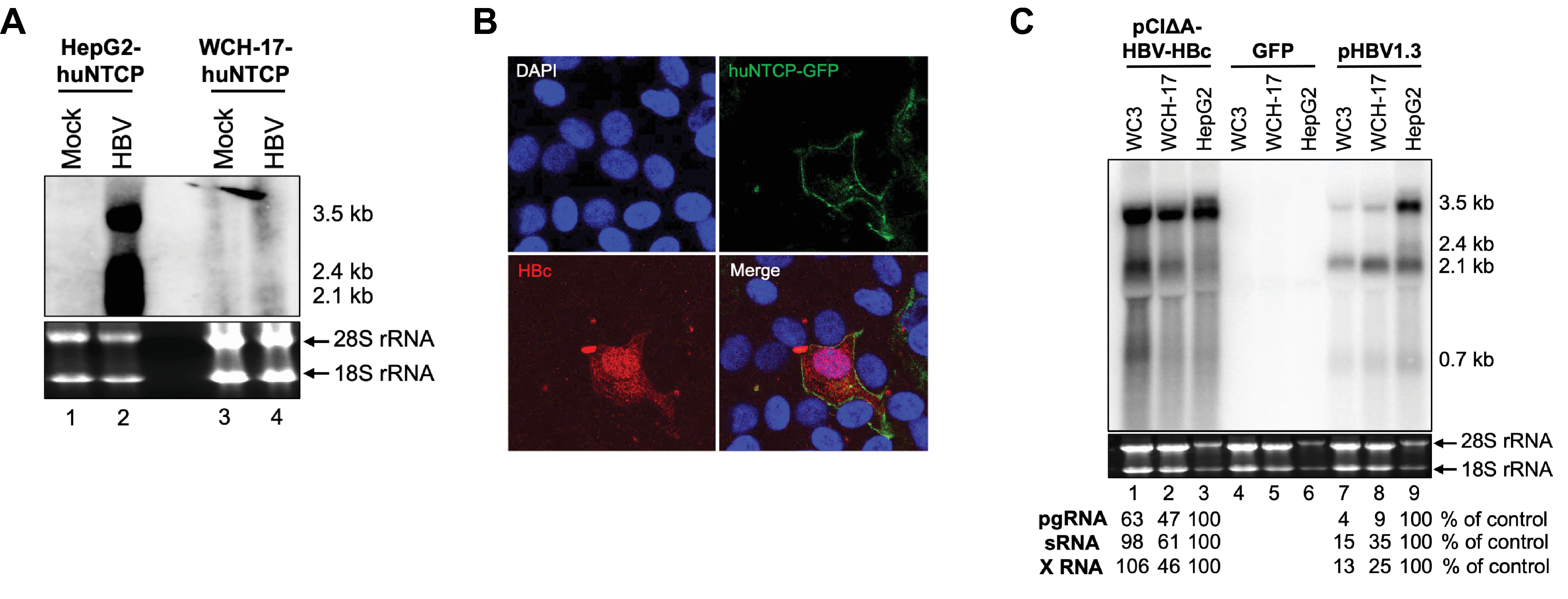

Supplement: S8 Fig — WCH-17-huNTCP and HepG2-huNTCP cells were infected with ca. 2000 genome equivalent (GE) HBV per cell. (A) Total RNA from mock- or HBV-infected WCH-17-huNTCP or HepG2-huNTCP cells was extracted at 8-days post-infection (dpi) and HBV RNAs were detected by northern blot analysis. Fifteen μg of total RNA from HepG2-huNTCP cells and 30 μg of total RNA from WCH-17-huNTCP cells were loaded. (B) Immunofluorescence analysis of HBc expression in HBV-infected WCH-17-huNTCP cells at 8 dpi (magnification 400X). (C) Woodchuck hepatic cell lines WC3 and WCH-17 and human hepatoma cell line HepG2 were transfected with the HBV replicon (pCIΔA-HBV-HBc or pHBV1.3) or control (GFP) plasmid. The cells were harvested at 5 days post-transfection. Total RNA from the transfected cells were extracted and HBV RNAs were detected by northern blot analysis. Ten μg of total RNA from WC3 and WCH-17 cells and 5 μg of total RNA from HepG2 cells were loaded. The relative levels of the various HBV RNA species in the different cell lines are indicated at the bottom, after normalizing to the levels of the respective RNA species in the transfected HepG2 cells that are set at 100%. (TIF) [file ppat.1010633.s008.tif]

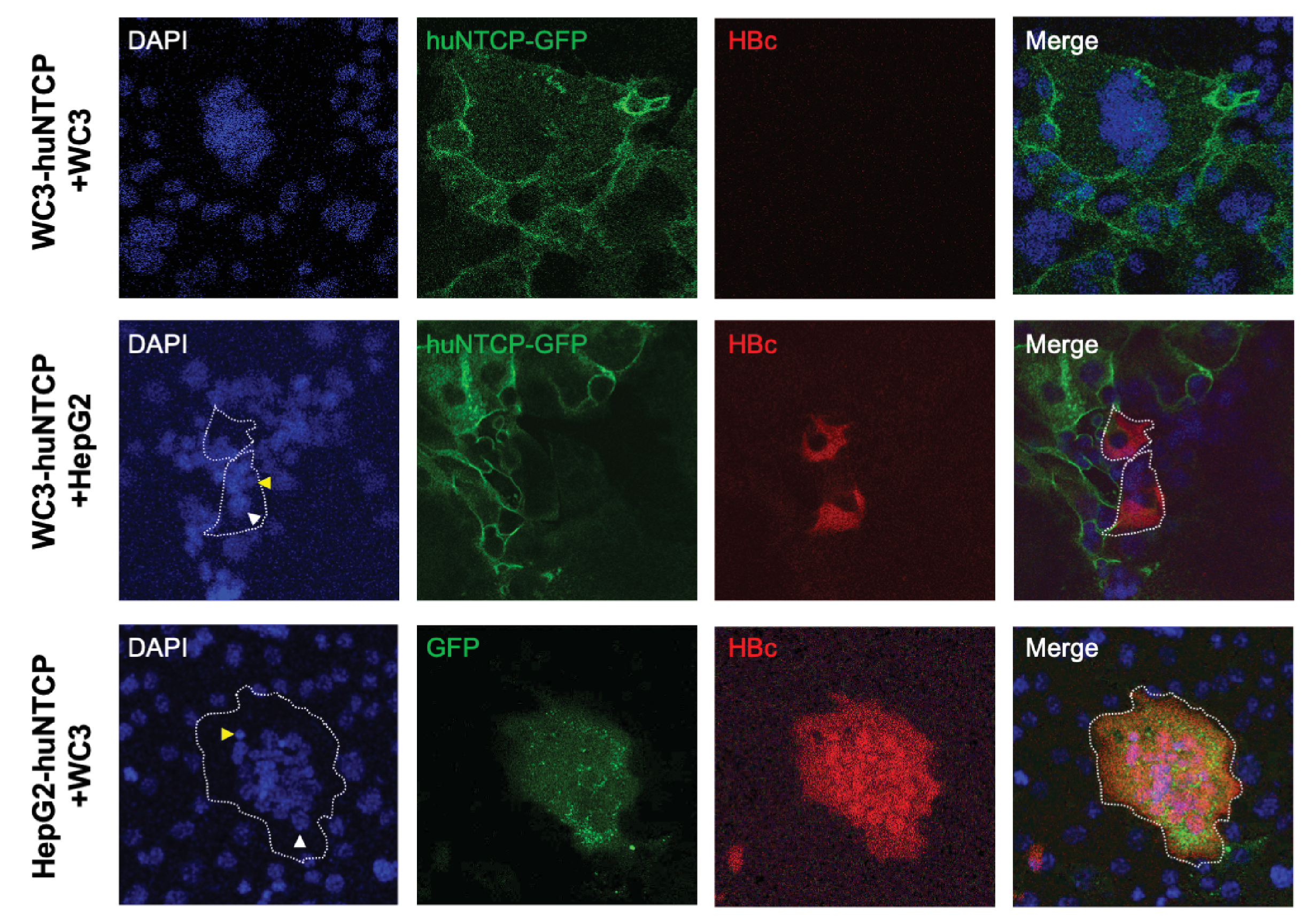

Supplement: S9 Fig — WC3 or HepG2 cells were co-seeded with WC3-huNTCP cells for 16 hours in a 24-well plate and treated with 50% PEG 1500 for 5 min to induce fusion. Similarly, WC3 cells were co-seeded with HepG2-huNTCP cells and fusion was induced. At 8 hours post-fusion, the cells were infected with HBV at a MOI of 2000 GE/cell. Immunofluorescence analysis of HBc expression in heterokaryotic cells was performed at 7 days post-infection (magnification 400X). The white and yellow arrowheads indicate the nucleus from the WC3 (or WC3-huNTCP) and HepG2 (or HepG2-huNTCP) cells, respectively. Also note that the GFP signal is diffuse throughout the cell in the HepG2-huNTCP cells as it was expressed as a free protein via an internal ribosomal entry site from the same RNA expressing huNTCP [63] whereas it is mostly on the plasma membrane in the WC3-huNTCP cells as it was expressed as the huNTCP-GFP fusion protein. (TIF) [file ppat.1010633.s009.tif]

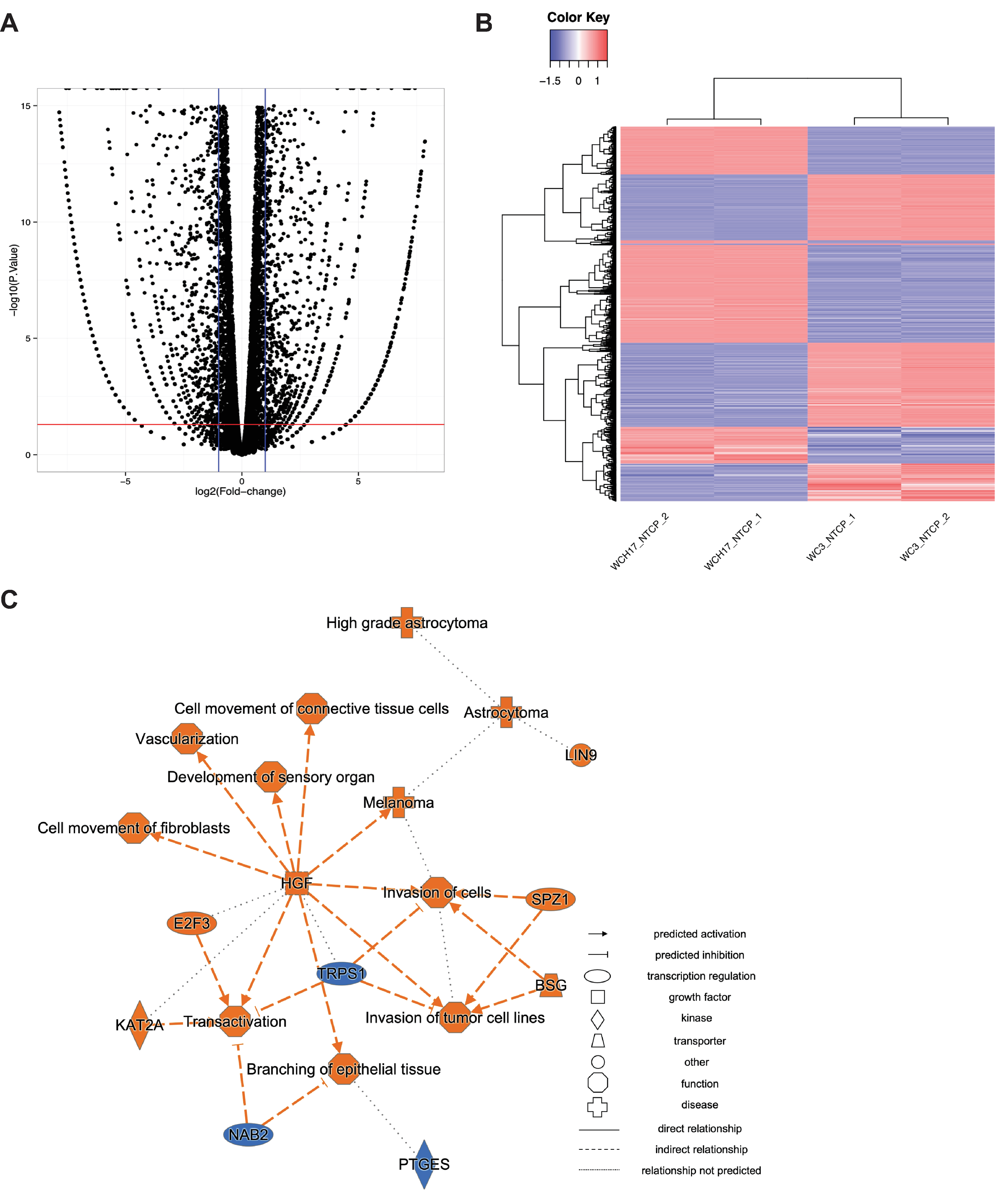

Supplement: S10 Fig — (A) Volcano plots of differentially expressed genes (DEGs) between WCH-17-huNTCP and WC3-huNTCP cells. (B) Heat map of DEGs between WCH-17-huNTCP and WC3-huNTCP (fold-change > 2, adjust p value < 0.05). (C) Graphic summary of IPA analysis of DEGs between WCH-17-huNTCP and WC3-huNTCP cells. (TIF) [file ppat.1010633.s010.tif]

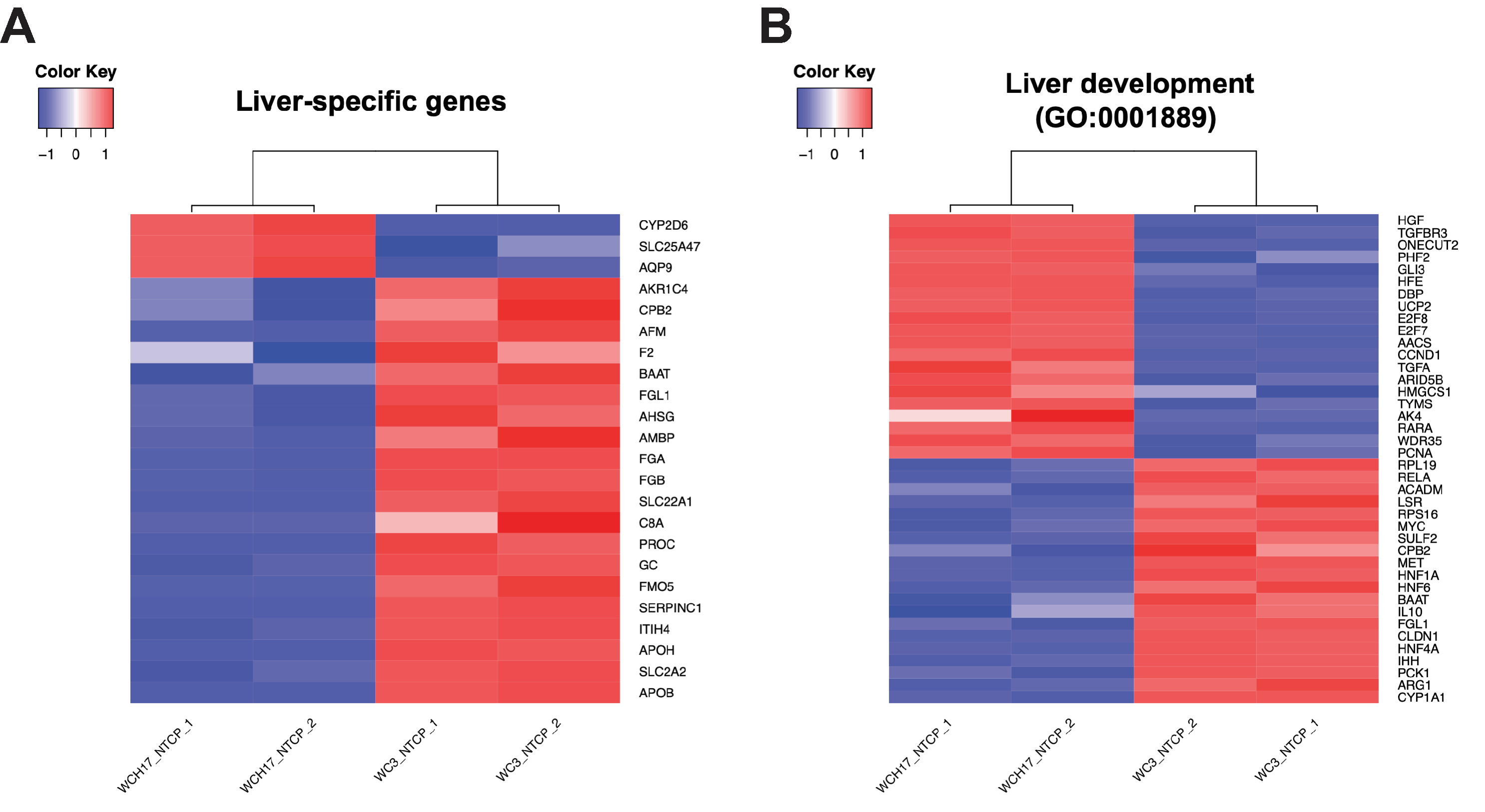

Supplement: S11 Fig — (A) Heat map of liver-specific genes that were differentially expressed between WC3 and WCH-17 huNTCP-expressing cells. (B) Heat map of DEGs associated with liver development (GO: 0001889). (TIF) [file ppat.1010633.s011.tif]

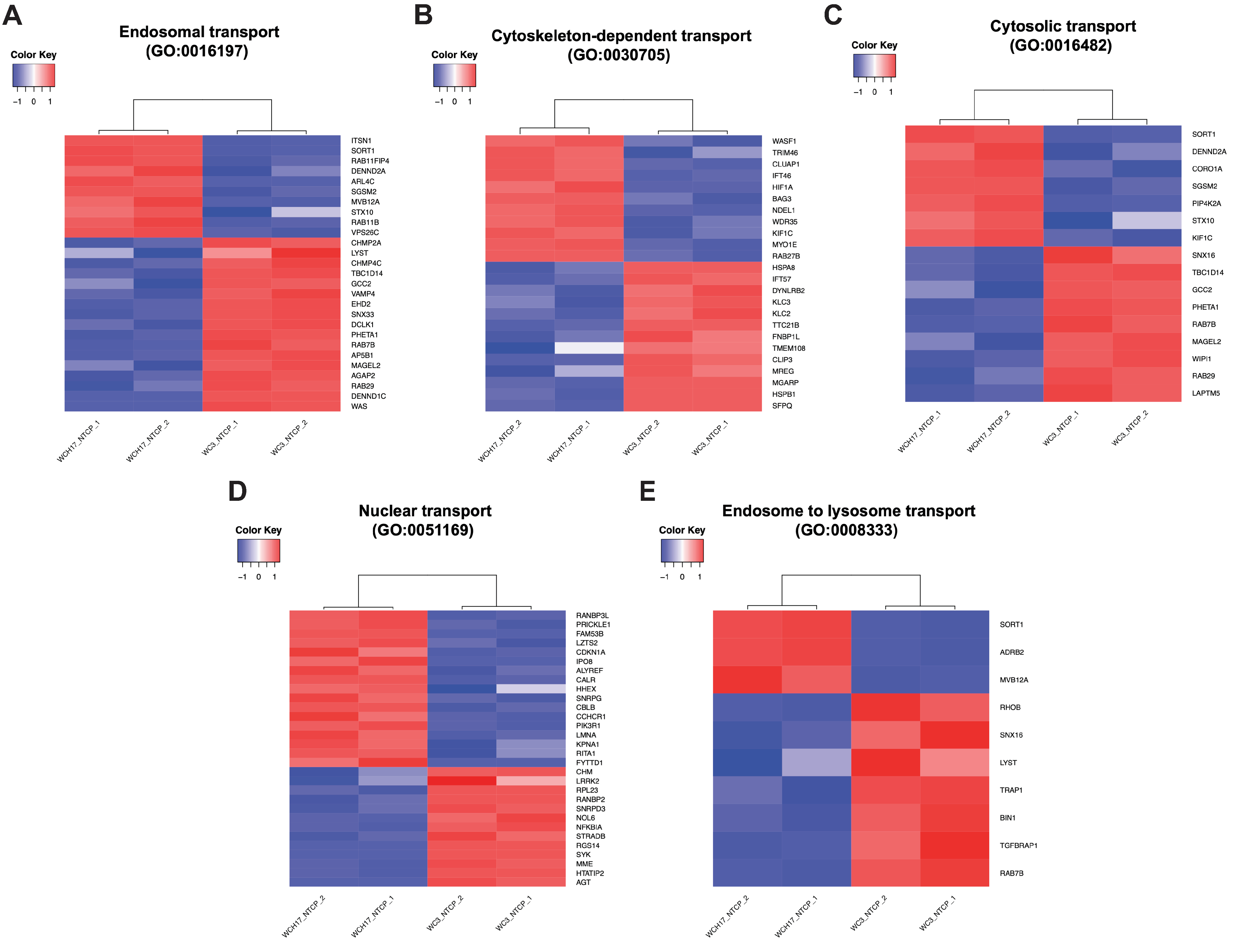

Supplement: S12 Fig — Heat map of genes differentially expressed between WC3 and WCH-17 huNTCP-expressing cells that are associated with (A) endosomal transport (GO: 0016197), (B) cytoskeleton-dependent transport (GO: 0030705), (C) cytosolic transport (GO: 0016482), (D) nuclear transport (GO: 0051169), and (E) endosome to lysosome transport (GO: 0008333). (TIF) [file ppat.1010633.s012.tif]
